# Imaging and quantitative analysis of integrin-dependent cell-matrix adhesions

**DOI:** 10.1016/j.xpro.2023.102473

**Published:** 2023-08-23

**Authors:** Anne-Marieke D. van Stalborch, Andrew G. Clark, Arnoud Sonnenberg, Coert Margadant

**Affiliations:** 1 Amsterdam University Medical Center, 1066 CX Amsterdam, the Netherlands; 2 Institute of Cell Biology and Immunology, Stuttgart Research Center Systems Biology, University of Stuttgart, 70569 Stuttgart, Germany; 3 Center for Personalized Medicine, University of Tübingen, Tübingen, Germany; 4 The Netherlands Cancer Institute, 1066 CX Amsterdam, the Netherlands; 5 Institute of Biology, Leiden University, 2333 BE Leiden, the Netherlands

**Keywords:** Cell Biology, Cell Culture, Microscopy

## Abstract

Integrin-dependent cell-extracellular matrix adhesion is essential for wound healing, embryonic development, immunity, and tissue organization. Here, we present a protocol for the imaging and quantitative analysis of integrin-dependent cell-matrix adhesions. We describe steps for cell culture; virus preparation; lentiviral transduction; imaging with widefield, confocal, and total internal reflection fluorescence microscopy; and using a script for their quantitative analysis. We then detail procedures for analyzing adhesion dynamics by live-cell imaging and fluorescence recovery after photobleaching (FRAP).

For complete details on the use and execution of this protocol, please refer to Margadant et al. (2012),^1^ van der Bijl et al. (2020),^2^ Amado-Azevedo et al. (2021).^3^

## Highlights

• Confocal microscopy of cell-matrix adhesions and analysis of integrin traffic
• Macro for automated quantitative analysis of cell-matrix adhesions
• Live imaging of adhesion dynamics by widefield, confocal, and TIRF microscopy
• Imaging and analysis of hemidesmosome behavior by FRAP

## Abstract (editor highlights)

Publisher’s note: Undertaking any experimental protocol requires adherence to local institutional guidelines for laboratory safety and ethics.

## Graphical abstract

**Figure:**
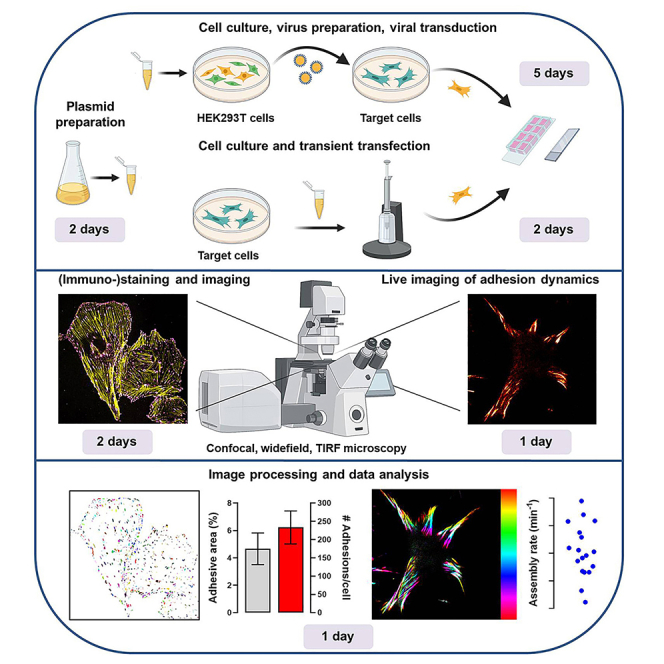

## Before you begin

### Experimental design considerations

**Figure 1:**
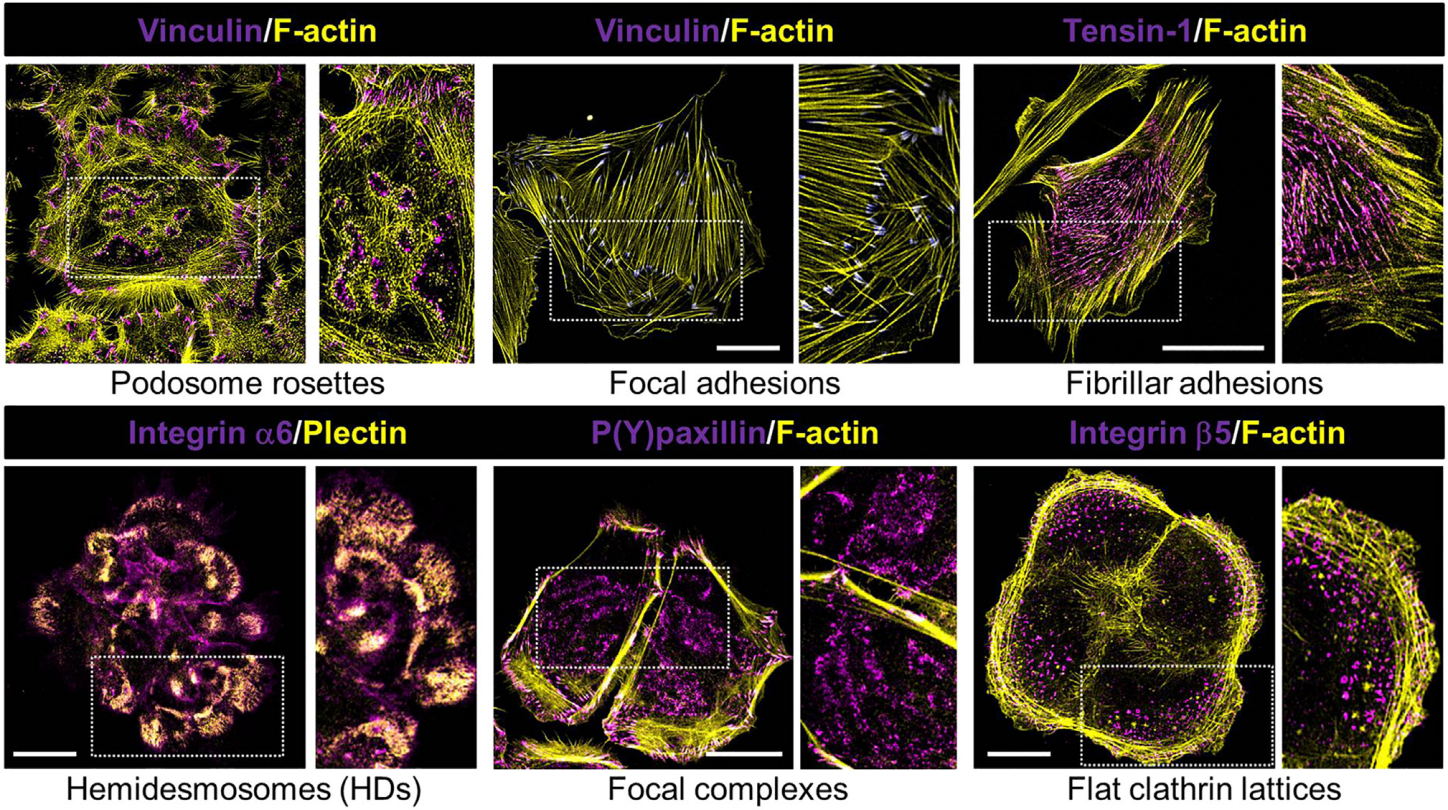
Integrin-dependent cell-matrix adhesions Images were acquired on a Leica SP8 confocal microscope as described in confocal imaging of cell-matrix adhesions in fixed cells. *Top,* podosomes in PA-JEB/β4 keratinocytes, FAs in REF-52 fibroblasts, and FBs in GDβ1 fibroblasts; *Bottom,* HDs , focal complexes, and flat clathrin lattices in PA-JEB/β4 keratinocytes. All images show antibody labelings (apart from tensin-1, which is ectopic expression of GFP-tensin-1) to mark the distinct adhesion structures (pseudocolored *magenta*), co-stained with cytoskeletal proteins F-actin or plectin (pseudocolored *yellow*). P(Y)paxillin, phospho(Y118)paxillin. Bars, 40 μm.

**Figure 2:**
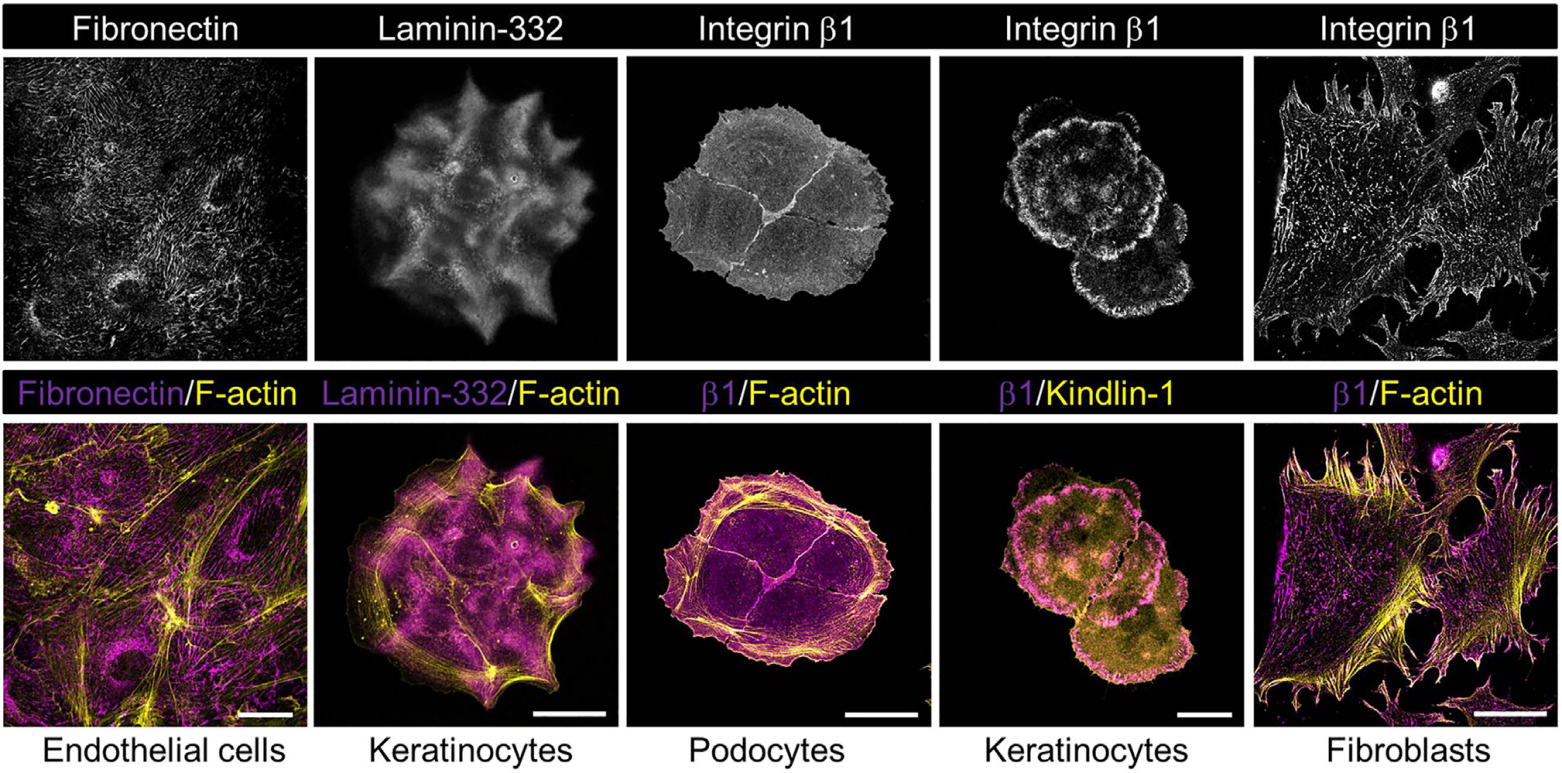
ECM deposition and distribution of β1 integrins (*Upper panel*) Confocal images (acquired on a Leica SP8 set-up as described in confocal imaging of cell-matrix adhesions in fixed cells) of endogenous intracellular FN in HUVECs and FN fibrils, laminin-332 deposited by murine MKα3 keratinocytes, and localization and distribution of β1 integrins in murine podocytes, human PA-JEB/β4 keratinocytes, and murine GDβ1 fibroblasts. (*Lower panel*) images from top panel (pseudocolored *magenta*) merged with F-actin or eGFP-kindlin-1 (pseudocolored *yellow*). Bars, 30 μm.

The current protocols are designed to visualize and analyze integrin-dependent cell adhesions to the extracellular matrix (ECM) using widefield, confocal, and total internal reflection fluorescence (TIRF) microscopy. We present protocols for the quantification of adhesions in fixed cells by antibody labeling, as well as imaging of adhesions in living cells expressing fluorescently tagged proteins. There are 24 different human integrins, which together recognize a wide range of matrix proteins including collagens, laminins, fibronectin (FN), and many others. Several different types of cell-ECM adhesions are known, including podosomes and invadopodia, focal complexes, focal adhesions (FAs) and fibrillar adhesions (FBs), flat clathrin lattices/reticular adhesions, and hemidesmosomes (HDs), which differ widely in size and appearance (Figure 1). The type of adhesion complex formed depends on cell type, cellular tension, the relative abundance of specific integrins and their dynamic behavior, and the available integrin ligand(s). Therefore, antibody labeling can result in very different patterns depending on cell type and culture conditions. For instance, β1 integrins organize into rings or cloud-like structures in keratinocytes, which form on patches of deposited laminin-332 (Figures 1 and 2). In contrast, β1 integrins have a diffuse membrane distribution in podocytes (which do not deposit laminin-332), and are localized in FAs and FBs, as well as in endosomes in fibroblasts (Figure 2). As another example, keratinocytes assemble vinculin-containing focal complexes and podosomes, whereas FAs are the major vinculin-containing adhesion structures in fibroblasts (Figure 1).

Different antibodies against the same protein may also result in different labeling patterns, for instance because they recognize their target only when in complex with another protein, or because they recognize a conformational epitope that is not always accessible. It is also noteworthy that many cells synthesize and deposit their own matrix, which greatly affects the distribution of ligated integrins. Examples include the distribution of keratinocyte integrins α3β1 and α6β4 on deposited laminin-332, or the organization of secreted FN into fibrils by integrin α5β1 on endothelial and mesenchymal cells (Figures 1 and 2). Finally, integrin ligands including FN, fibrinogen, and vitronectin are abundant in serum and are incorporated by cells into the matrix. Thus, depending on the presence of serum and culture time, adhesion complexes may reflect merely the ligation of integrins on newly assembled matrix, rather than the matrix that was used to coat the culture surface.

The quality of immunofluorescence images acquired with antibody labelings will further strongly depend on the degree of non-specific binding, as well as the extent of clustering of a given protein and its localization outside of cell-matrix adhesions, for instance because there is a strong cytoplasmic localization. In the latter case, the image signal-to-noise ratio can be improved using TIRF microscopy, which restricts imaging to within 200 nm of the coverslip and thereby reduces out-of-focus fluorescence. TIRF is also ideal for live cell imaging of adhesion dynamics using fluorescent proteins, however it is also very sensitive to small fluctuations in air current or temperature, potentially causing focal drift. For very flat cells with a low cytoplasmic volume above the basal surface, or when the protein under investigation is strongly enriched in adhesions versus the cytoplasm, TIRF is not required and confocal or even widefield microscopy can suffice. For any live-cell experiment with cells expressing fluorescent proteins, it is important to optimize conditions to reduce phototoxicity and bleaching, and to select cells with low expression levels, as too high expression may cause artefacts. Because each individual experiment has specific requirements on experimental design, we also discuss some alternatives and adjustments to the protocol and set-up described here.

### Prepare buffers, cell culture media, and substrates

**Timing: ∼4 h**

1. Prepare mammalian cell culture media as described in materials and equipment.
2. Prepare required solutions as described in materials and equipment.
3. Prepare substrates for tissue culture and imaging.
  a. Clean and sterilize coverslips of the desired diameter by washing with 70% EtOH.
  b. Put clean and sterile coverslips in a well plate.
  c. Wash coverslips with PBS.
  d. If desired, coat coverslips and other cell culture substrates with collagen-I (Col-I; 3 μg/mL), FN (5 μg/mL), or gelatin (0.1%), dissolved in PBS.
  e. Wash the coated substrates twice with PBS.

***Note:*** Coated coverslips can be kept at 4°C for up to two weeks.

***Note:*** Required concentrations of used matrix proteins differ between cell types and conditions. It is recommended to first determine the optimal concentration for each matrix protein.

***Note:*** Endothelial cell adhesion to tissue cultures surfaces is promoted by coating with gelatin (a denatured and hydrolyzed from of collagen), while keratinocytes and other epithelial cells generally adhere well to Col-I. Adhesion of many cell types, including endothelial and mesenchymal cells, is supported by FN. Col-I and gelatin are very sticky and coat efficiently already within 10 min at 37°C, but FN requires longer coating (1–2 h). Human embryonic kidney (HEK)293T cells, used for production of lentiviral particles, do not always adhere efficiently to non-coated surfaces, and their adhesion is promoted by coating with FN. Alternatively, culture surfaces for HEK cells may be coated with fetal calf serum (FCS), which contains high amounts of vitronectin and FN, for 1–2 h at 37°C. We routinely re-use FCS and FN solutions for coating three times (troubleshooting 1).

***Note:*** Different ECM proteins such as FN, laminins, or collagens can have very different effects on migratory behavior, cell morphology, and the size, number, and organization of cell-matrix adhesions, reflecting the engagement of different integrins (troubleshooting 2).

***Note:*** Recombinant laminins are expensive. As an alternative, a cell-derived matrix rich in laminins can be used. We used here the Rac-11P murine mammary cell line which secretes copious amounts of laminin-332.^4^ Alternatively, Engelbreth-Holm-Swarm (EHS) mouse sarcoma cells can be used.^5^ Rac-11P cells are grown to confluency in the appropriate culture dish and then incubated with 10 mM EDTA in PBS, supplemented with protease inhibitors (for example the protease inhibitor cocktail from Abcam), for 18 h at 4°C. By vigorous pipetting the next day, the cells are detached (often as a single sheet), exposing the laminin-rich matrix.

### Cell culture and passage

**Timing: 2–3 days**

4. Grow cells in T75 flasks in the appropriate medium at 37°C and 5% CO_2_.

**CRITICAL:** While calcium is required for cellular processes including cell-cell adhesion via cadherins, excessive calcium can block proliferation of several cell types such as keratinocytes. Therefore, calcium amounts in culture media for keratinocytes should be < 100 μM. However, for imaging of HDs in keratinocytes, switching from keratinocyte growth medium (low calcium) to DMEM/FCS (high calcium) is recommended, because it triggers the assembly of these structures.

5. To passage cells, aspirate the medium and wash the cells with 37°C PBS.
6. Add trypsin/EDTA in PBS (∼1 mL for a T75 flask) and distribute over the entire surface.
7. Place cells back at 37°C and 5% CO_2_. The time required for trypsin-induced cell detachment differs between cell types and the matrix they grow on but is roughly between 1 and 20 min.
8. Confirm that cells have rounded up and/or detached using a tissue culture tabletop microscope.

***Note:*** We have used an inverted Zeiss Axiovert 25 microscope at 10× (NA 0.25) or 20× (NA 0.3) magnification, connected to a Zeiss CCD camera (Axiocam MRC) and Zeiss Mr. Grab 1.0 software to acquire images. Several other options instead of the set-up used here are available.

9. Gently tap flask to fully detach rounded cells.
10. Resuspend the cells in culture medium and transfer to a new culture dish.

***Note:*** Resuspension of trypsinized cells in cell culture media with a high amount of FCS (at least 2%) will inactivate the trypsin. In media with lower amounts of FCS, such as the KGM described here, trypsinized cells are resuspended in medium and then centrifuged, whereafter supernatant containing the trypsin is discarded. Alternatively, Trypsin Neutralization Solution can be used, in a 1:1 ratio with the used trypsin before resuspending the cells. This will also prevent Trypsin from digesting present growth factors.

***Note:*** Cell culture substrates for continuous culture (e.g., T75 flasks) can generally be re-used 2-3 times, however be aware that some, if not all, cells deposit huge amounts of matrix proteins onto the surface which will only be partially cut by trypsin, and may have strong effects on cell phenotype, behavior, proliferation, and/or differentiation. Furthermore, re-use of flasks may increase the risk of contamination.

***Note:*** We use commercially available pools of human umbilical vein endothelial cells (HUVECs), which we expand to the extent that the same batch of cells can be used for a large number of conditions during consecutive experiments.^6^^,^^7^

***Note:*** Maintaining constant cell culture conditions will enhance the reproducibility of results. For most cell types, it is preferred to use cells that have not been maintained in culture for too long (generally, less than 10 passages for primary cells and less than 25 passages for immortalized cells would be recommended).

### Production of retroviral particles for transduction

**Timing: 7 days**

This method describes how to produce retroviral particles for the transduction of target cells. If lentiviral transduction is preferred, omit steps 11-35 and proceed with step 36. Retroviral plasmids containing long-terminal repeats are grown in Recombinase-deficient bacterial strains, such as *Escherichia coli* Stbl3, in Lennox Broth containing selection marker (e.g., ampicillin, 100 μg/mL) at 30°C for 16–20 h. Plasmid DNA is then purified according to the NucleoBond Xtra protocols for Mini, Midi, or Maxi preps (Macherey-Nagel), using DNA purification kits (Qiagen).

11. Culture Phoenix cells in DMEM/FCS as described in steps 1–7 of cell culture and passage.

***Note:*** Phoenix cells are a derivative of HEK293T cells that can be used to produce retroviral particles upon transfection with a retroviral vector such as LZRS, encoding the protein of interest. For the transduction of human cells with these particles, amphotrophic Phoenix (Phoenix-AMPHO) expressing an amphotropic envelope protein should be used, while for the transduction of other non-human mammalian target cells, ecotrophic Phoenix (Phoenix-ECO) can be used.

***Note:*** It is important to make single-cell suspensions after trypsinization. If cells are not properly suspended and seeded in clumps, this will negatively affect transfection.

12. Seed cells in a 10 cm diameter dish in 8 mL DMEM/FCS at approximately 30%–40% confluency and incubate at 37°C and 5% CO_2_.
13. After 1 day, the cells can be transfected. Cells should be approximately 60%–80% confluent. In the flow cabinet, mix 10 μg of DNA with 50 μL of 2.5 M CaCl_2_ and fill up to 500 μL with distilled H_2_O.
14. Vortex the DNA/CaCl_2_ mixture while slowly and dropwise adding 500 μL of 2× Hepes-Buffered Saline (HBS). A co-precipitate of DNA and calcium phosphate will form.
15. Leave the mixture for max 20 min at 20°C in the flow cabinet.
16. Distribute the co-precipitate carefully and evenly over the dish and gently swirl to mix the DNA/calcium phosphate complexes with medium.

***Note:*** This step should be done carefully as Phoenix cells are loosely adherent and may detach.

17. Incubate the cells at 37°C and 5% CO_2_ for 6–16 h.
18. Replace the medium very carefully with 8 mL of DMEM/FCS.
19. Harvest the culture medium 48 h after transfection, centrifuge at 500 × *g* for 5 min.
20. Filter the harvest with a 0.45 μm pore filter, aliquot and store at −80°C or proceed with transduction as described below.

***Note:*** If more supernatant is required, Phoenix cells can be placed back in the incubator with fresh medium for an additional round of harvesting the next day.

**CRITICAL:** It is required to perform steps 16-20 in a Biosafety Level (BSL)-2 laboratory. Adhere strictly to BSL-2 regulations on sample handling and waste disposal.

### Retroviral transduction followed by cell sorting for stable expression of integrins or fluorescent adhesion proteins

**Timing: 5–14 days**

This method describes how to generate cell lines (stably) expressing fluorescent adhesion proteins or integrins using retroviral transduction, followed by antibiotic selection and/or fluorescence-activated cell sorting of transduced cells. The latter approach can be used directly to sort cells that express a fluorescent protein, or following the labeling of stably expressed integrins on the surface using an antibody against the integrin extracellular domain. The cell lines stably expressing β1, β3, or β4 used here have been generated in this way.

21. Culture target cells in appropriate medium as described in steps 1–10 of cell culture and passage.
22. After 1 or 2 days, seed cells for retroviral transduction in 6-well plates (in 1 mL culture medium/well) at approximately 35% confluency, 1 or 2 wells per condition. Coat wells prior to cell seeding with appropriate ECM protein if necessary, as described in step 3 of prepare buffers, cell culture media, and substrates.
23. The following day, transduce cells with viral particles in DMEM/FCS, obtained as described in steps 11-20 of production of retroviral particles for transduction (1 mL/well of a 6-wells plate), in a 1:1 ratio with the culture medium.

***Note:*** Cells should be 60%–80% confluent.

***Note:*** Retroviruses can only infect actively dividing cells and therefore this approach is less suitable for the transduction of quiescent or poorly proliferating/dividing cells.

24. Incubate the cells for 12–16 h with the retroviral particles at 37°C and 5% CO_2_.
25. The next day, replace the medium containing retroviral particles with culture medium (3 mL/well) and incubate at 37°C and 5% CO_2_.

***Note:*** The efficiency of retroviral transduction can be enhanced using Polybrene, a cationic polymer that increases the interaction between cell membranes and viral particles.

26. The next day, replace the medium with cell culture medium containing zeocin (typically 50–400 μg/mL) to select positive cells and place at 37°C and 5% CO_2_.
27. Maintain cells under zeocin pressure, refreshing the medium every 2–3 days, and transfer the cells to a larger surface area (for instance a 10 cm diameter dish or T75 flask) when sufficient survivors are obtained.

***Note:*** Always take 1 well of non-transduced cells along to confirm zeocin-induced cell death. A kill curve should be performed first to establish the ideal zeocin concentration (giving 100% cell death after 3 days or 2 passages) for each cell type. Selection with zeocin is relatively slow and generally requires a couple of passages.

28. After 7–14 days, significant numbers of positive cells should be acquired.

***Note:*** The expression is now stable and cells can be used for experiments, further expanded to freeze vials for cryostorage, or processed for flow cytometry-based cell sorting as described in the following steps.

***Note:*** Flow cytometry can be used to enrich for positive cells or to select cells that have similar levels of expression of the introduced protein. It may be useful to exclude cells in which the expression is low, as this may have less biological effects than desired or will compromise the visibility of the protein by microscopy. Conversely, too high expression of the protein may lead to abnormal dynamics of adhesion complexes, artificial protein localization, and even protein aggregation in the cytosol or ER. Cells that have been transduced to express fluorescent proteins, such as eGFP-Kindlin-1 used here, can be sorted directly without labeling, while cells (over)expressing integrins can be sorted following prior labeling of cell-surface integrins with antibodies directed against their ectodomains.

29. Trypsinize cells as described in cell culture and passage. Cells expressing a fluorescent protein can now be used for cell sorting (step 35 below). For labeling of transduced integrins, follow steps 30-34 below.
30. Wash cells twice in PBS containing 2% FCS (to reduce nonspecific antibody binding).
31. Incubate with primary anti-integrin antibody for 30–45 min at 4°C. The cells used here engineered to express β1, β3, or β4 integrin were labeled with 1 μg/mL primary antibodies (clone TS2/16 for β1, clone C-17 for β3, and clone 439-9B for β4).
32. Wash cells twice in 2% FCS/PBS.
33. Incubate the cells with appropriate fluorophore-conjugated secondary antibodies for 45 min at 4°C.
34. Wash cells twice in 2% FCS/PBS.
35. Sort cells in PBS/0.5% FCS and collect them in PBS with 5% FCS. Here we used a MoFlo High Speed Cell Sorter (Beckman Coulter).

***Note:*** Primary antibodies that are directly conjugated to a fluorophore can also be used, in which case steps 33-34 are not required.

***Note:*** For imaging of individual cells, zeocin selection and/or cell sorting may not be required since for imaging experiments, positive cells can be selected by visual inspection. Nevertheless, the efficiency of retroviral transduction, although it depends on the cell type, is generally much lower than that of lentiviral transduction (see below) and therefore selection and/or sorting is recommended to enrich for positive cells.

### Production of lentiviral particles for transduction

**Timing: 5 days**

This method describes how to prepare lentiviral particles for efficient transduction of target cells to express fluorescent fusion proteins to image cell-matrix adhesions, or short hairpin (sh)RNAs to efficiently suppress the expression of a given target protein involved in the regulation of these adhesions. In contrast to retroviruses, lentiviruses also infect quiescent or poorly proliferating cells, yielding a much higher transduction efficiency. First, bacteria containing plasmid DNA, such as *Escherichia coli* DH5α, are inoculated in Lennox Broth containing selection marker (e.g., ampicillin, 100 μg/mL) and grown for 12–16 h at 37°C. Lentiviral plasmids containing long-terminal repeats are grown in a Recombinase-deficient bacterial strain, such as *Escherichia coli* Stbl3 at 30°C for 16–20 h. Plasmid DNA is then purified according to the NucleoBond Xtra protocols for Midi or Maxi preps (Macherey-Nagel) using DNA purification kits (Qiagen). Co-constructs needed for the production of lentiviral particles (Table 1) are purified from *Escherichia coli* Stbl3, grown at 30°C.

36. Seed HEK293T cells at approximately 30% confluency in T75 tissue culture flasks.
37. Cells should be ready for transfection the next day (40%–70% confluent). Transfect HEK293T cells with constructs as in Table 1.
38. Add 30 μL TransIT-LT1 to 750 μL Opti-MEM and mix thoroughly.
39. Make a transfection mix as indicated in Table 1.

***Note:*** Optimal plasmid concentration is 1000 ng/μL.

40. Let the transfection mix incubate at 20°C in the dark for 20 min.

**CRITICAL:** Do not leave the transfection mix longer than 30 min prior to use.

41. Incubate HEK293T cells with transfection mix in the incubator at 37°C and 5% CO_2_.
42. After 6 h, remove the transfection medium and add 10 mL of DMEM/FCS.
43. Incubate the cells at 37°C and 5% CO_2_.
44. Harvest the culture medium 24–48 h post-transfection, centrifuge at 500 × *g* for 5 min, and store at 4°C.
45. Add 10 mL of DMEM/FCS to the cells and place them back at 37°C and 5% CO_2_.
46. Harvest the culture medium 48–72 h after transfection, centrifuge at 500 × *g* for 5 min.
47. Mix the two harvests, filter with a 0.45 μm pore filter, aliquot and store at −80°C.

**CRITICAL:** It is required to perform steps 41-47 in a BSL-2 laboratory. Adhere strictly to BSL-2 regulations on sample handling and waste disposal.

**CRITICAL:** For efficient target cell transduction, especially when creating cell populations with stable knockdown of a gene of interest, a good viral titer is essential (troubleshooting 3).

**Table 1:** Transfection mix for the production of lentiviral particles in HEK293T cells

	Description	Amount
*pHDMG·G VSV ENV*	encodes the envelope protein of Vesicular Stomatitis Virus	4950 ng
*pHDM·HgpM2 GAG/POL*	encodes structural viral proteins and reverse transcriptase	1667 ng
*pRC-CMV-REV1B*	Encodes Rev1b, necessary for efficient synthesis of viral proteins	1667 ng
*pHDM-TAT1B*	encodes Tat1b, which facilitates viral entry into target cells	1667 ng
*pLKO.1-puro* with shRNA or lentiviral expression vector	encodes shRNA to suppress expression of target protein and resistance gene for puromycin selection encodes fluorescent protein of interest and resistance gene for possible selection	21667 ng
*Trans*IT-LT1	Transfection reagens	30 μL
Opti-MEM	Medium	750 μL

### Lentiviral transduction for stable expression of fluorescent adhesion proteins or shRNAs

**Timing: 5–6 days**

This method describes a lentiviral transduction procedure for stable expression of fluorescently labeled adhesion proteins or shRNAs for suppression of target gene expression by RNA interference.

48. Culture target cells in appropriate medium as described in cell culture and passage.
49. After 1 or 2 days, seed cells for lentiviral transduction in 6-well plates at 40%–60% confluency, 1 or 2 wells per condition. Coat wells prior to cell seeding with appropriate ECM protein if necessary, as described in step 3 of prepare buffers, cell culture media, and substrates.
50. The following day, transduce cells with viral particles, obtained as described in steps 36-47 of production of lentiviral particles for transduction.
51. Incubate the cells for 12–16 h with the lentiviral particles (1 mL/well of a 6-wells plate) in DMEM/FCS.

***Note:*** Supplement with culture medium, depending on virus concentration and/or efficiency. It is advised to use as little virus as possible as high viral titers may lead to adverse effects such as cell death. Also, expression of the target gene should not be too high as this may lead to altered adhesion dynamics or artificial localization and even aggregation of fluorescent proteins in the cytosol or ER. To begin with, titration of the virus, followed by selection with puromycin, is recommended to determine the lowest viral titer that yields efficient transduction.

52. Replace the medium with cell culture medium containing puromycin (1–5 μg/mL) to select positive cells.
53. After 2–3 days, selection should be finished. The expression is now stable and cells can be frozen for cryostorage to maintain back-up.

**CRITICAL:** Protocol steps 50-53 must be performed in a BSL-2 laboratory. Tight adherence to BSL-2 regulations on sample handling and the disposal of plastic ware and media is required.

***Note:*** Always take 1 well of non-transduced cells along to confirm puromycin-induced cell death. A kill curve should be performed first to establish the ideal puromycin concentration (giving 100% cell death after 3 days or 2 passages) for each cell type.

***Note:*** While lentiviral transduction is a fast and efficient way for gene delivery, the size limit of lentiviral vectors prohibits the packaging of very large proteins, in particular when fused to a fluorescent protein.

***Note:*** For imaging of individual cells, puromycin selection is not absolutely necessary since the efficiency of lentiviral transduction is generally high and positive cells can be easily selected by eye for imaging.

***Note:*** Other methods of delivery such as retroviral transduction and use of other selection markers is also possible, but lentiviral transduction followed by puromycin selection is most efficient and fastest for most cell types.

### Transient expression of fluorescent proteins in cells with stable gene suppression

**Timing: 2–3 days**

While lentiviral or retroviral transduction to deliver fluorescent proteins has certain advantages as described above, it may sometimes be more convenient to use a transient transfection procedure, for instance when cells have already been transduced and selected with antibiotics. In this method we describe lentiviral transduction in HUVECs for stable suppression of protein expression (in this case of Arg/Abl2), followed by transient transfection with GFP-tensin-1 to visualize FBs, which are important adhesion structures in endothelial cells associated with FN fibrils.^3^^,^^8^

54. Transduce and select HUVECs as described in lentiviral transduction for stable expression of fluorescent adhesion proteins or shRNAs.

**CRITICAL:** A scrambled sequence in pLKO.1 should be taken along in parallel as a control.

***Note:*** It is advised to use more than 1 shRNA against the target of interest to avoid erroneous interpretation of results due to potential off-target effects.

**CRITICAL:** Although too high viral titers may be detrimental to cell health, the titer should be high enough to ensure that sufficient transduced cells are obtained after selection (troubleshooting 3).

55. For transient transfection by electroporation, use approximately 2 μg DNA for 3–5 × 10^5^ cells per transfection with a 100 μL microporation tip.
56. Place buffer R and E2 at 20°C for 2 h before the microporation.
57. Prepare culture plates with appropriate coating by filling wells with pre-warmed endothelial growth medium (EGM-2).
58. Fill microporation tube with buffer E2 (3 mL). Place tube in the pipette station with electrodes facing each other. Fully insert the tube until you hear a “click” sound.
59. Use the following settings for HUVECs: voltage = 1300 V, pulse width = 30 ms, pulse = 1.

***Note:*** While these settings are optimal for HUVECs, other cell types may require different settings. Optimize for other cell types.

60. Prepare cell suspension by trypsinization and centrifugation at 500 × *g* for 3 min, and resuspend cell pellet in 120 μL buffer R, then mix with DNA.
61. Place a 100 μL microporation tip on the microporation pipette by fully pressing in the push button while fitting the tip on the pipette.
62. Aspirate the cell/DNA sample using the microporation pipette.
63. Place the pipette into the microporation tube/station with the metal head of the pipette facing the side electrode in the microporation tube. Insert the pipette vertically until you hear a “click” sound.

***Note:*** Make sure to remove any air bubbles from within the tip. Air bubbles make an electric disconnection and may generate an arc during the microporation, leading to lower transfection efficiency and cell viability.

64. Press the start button to deliver the electric pulse.
65. Transfer cell suspension after microporation to culture wells containing pre-warmed EGM-2 and place in the incubator at 37°C and 5% CO_2_.

***Note:*** In general, expression is optimal 24–48 h after microporation.

## Key resources table

**Table:** 

REAGENT or RESOURCE	SOURCE	IDENTIFIER
**Antibodies**		

Mouse anti-FN (clone 10) for immunofluorescence (1:500)	BD Transduction Laboratories	Cat#610077
Mouse anti-human integrin β1 (clone TS2/16) for cell sorting and trafficking experiments (1 μg/mL)	A kind gift from Dr. T. Springer	
Rat anti-mouse integrin β1 (clone MB1.2) for immunofluorescence (1:100)	A kind gift from Dr. B. Chan	
Mouse anti-human integrin β3 (clone C-17) for cell sorting (1 μg/mL)	A kind gift from Dr. E. van der Schoot	
Mouse anti-human integrin β3 (clone 23C6) for immunofluorescence (1:100)	Thermo Fisher Scientific	Cat#11-0519-42
Rat anti-human integrin β4 (clone 439-9B) for cell sorting and trafficking experiments (1 μg/mL)	A kind gift from Dr. S.J. Kennel	
Rabbit anti-human integrin β5 (EM09902) for immunofluorescence (1:200)	A kind gift from Dr. S. Goodman	Goodman et al.^9^
Rat anti-mouse/human integrin α6 (clone GoH3) for immunofluorescence (1:5 from hybridoma supernatant)	Home made	Sonnenberg et al.^10^
Rabbit anti-laminin-332 (R14) for immunofluorescence (1:500)	A kind gift from Dr. M. Aumailley	
Mouse anti-paxillin (clone 5H11) for immunofluorescence (1:500)	Thermo Fisher Scientific	Cat#AHO0492
Mouse anti-human plectin (HD1) for immunofluorescence (1:50)	A kind gift from Dr. K. Owaribe	
Rabbit anti-phospho(Y118)paxillin for immunofluorescence (1:100)	Abcam	Cat#ab4833
Mouse anti-phosphotyrosines (clone 4G10) for immunofluorescence (1:5 from hybridoma supernatant)	A kind gift from Dr. K. Wilhemsen	
Mouse anti-vinculin (clone hVIN-1) for immunofluorescence (1:100)	Sigma-Aldrich	Cat#V9264
Anti-mouse, rat, and rabbit secondary antibodies conjugated to appropriate fluorophores for cell sorting and immunofluorescence (1:200)	Thermo Fisher Scientific	e.g., Cat#A11001, Cat#A11006, Cat#A21244

**Bacterial and virus strains**		

*Escherichia coli* DH5α	New England Biolabs	Cat#C29871
*Escherichia coli* Stbl3	Invitrogen	Cat#C737303

**Chemicals, peptides, and recombinant proteins**		

Ampicillin	Merck	Cat#59349
Bovine pituitary extract	Lonza	Cat#CC-4009
CaCl_2_.2H_2_O	Merck	CAS 10035-04-08
Collagen-I (Col-I)	Advanced BioMatrix	Cat#5005
Dabco 33-LV	Sigma-Aldrich	Cat#290734
D-Glucose	Sigma-Aldrich	CAS 50-99-7
Dimethyl sulfoxide (DMSO)	J.T.Baker	CAS 67-68-5
Dulbecco’s Modified Eagle Medium (DMEM)	Thermo Fisher Scientific	Cat#41965-039
EDTA Triplex III	Merck	CAS 6381-92-6
Endothelial Basal Cell Growth Medium-2 (EBM-2)	PromoCell	C-22211
Endothelial Growth Medium 2 Kit	PromoCell	C-22111
Epidermal growth factor (EGF)	Gibco	PHG0311
FCS, heat-inactivated	Bodinco	5010
FN	Sigma-Aldrich	Cat#F1141
Gelatin	Sigma-Aldrich	Cat#G1890
L-Glutamine	Sigma-Aldrich	Cat#G7513
Glycerol	Merck	Cat#77067
Glycine	Sigma-Aldrich	Cat#G7126
HCl	Merck	Cat#258148
HEPES (free acid)	Sigma-Aldrich	Cat#391338
Hoechst 33342	Thermo Fisher Scientific	Cat#H3570
KCl	Sigma-Aldrich	P5405
Keratinocyte serum-free medium	Gibco	Cat#17-005-042
Lennox Broth	Sigma-Aldrich	Cat#L3022
MeOH	Merck	Cat#1060352500
MgCl_2_.6H_2_O	Sigma-Aldrich	CAS 7791-18-6
NaOH	Merck	Cat#S5881
NaCl	Sigma-Aldrich	Cat#S9888
Na_2_HPO_4_	Sigma-Aldrich	Cat#S9763
Opti-MEM Reduced Serum Medium, GlutaMAX supplement	Thermo Fisher Scientific	Cat#51985034
Paraformaldehyde (PFA)	Merck	Cat#1.04005
Penicillin/Streptomycin	Sigma-Aldrich	P0781
Phalloidin, Texas Red-conjugated	Thermo Fisher Scientific	Cat#T7471
Phosphate-buffered saline (PBS), pH 7.0	Fresenius Kabi	N/A
Mowiol 4-88	Sigma-Aldrich-Merck	Cat#81381
Neon Transfection System 10 μL kit	Invitrogen	Cat#MPK1096
Polyethylene glycol (PEG) 6000	Sigma-Aldrich	CAS 25322-68-3
Protease inhibitor cocktail	Abcam	Cat#ab271306
Puromycin	InvivoGen	ant-pr-1
Sodium pyruvate	Thermo Fisher Scientific	Cat#11360070
Thrombin	Sigma-Aldrich	Cat#T1063
*Trans*IT-LT1	Mirus Bio	MIR2300
Triton X-100	Sigma-Aldrich	Cat#T8787
Trypsin	Sigma-Aldrich	Cat#59418C
Trypsin Neutralization Solution	Lonza	Cat#CC-5002
Zeocin	Gibco	Cat#R25001

**Experimental models: cell lines**		

HEK293T cells	ATCC	CRL-3216
Human epidermoid carcinoma A431 cells	ATCC	CRL-1555
HUVECs	Lonza	C2519A
Human pyloric atresia-junctional epidermolysis bullosa (PA-JEB) cells expressing β4-GFP		^11^^,^^12^
Murine β1−/− GE11 cells reconstituted with wild-type or mutant β1 or overexpressing wild-type or mutant β3 (GEβ1 and GEβ1^Y795A^)		^1^^,^^2^^,^^13^^,^^14^^,^^15^^,^^16^
Murine podocytes		^17^
Murine β1−/− GD25 cells reconstituted with β1 (GDβ1)		^1^^,^^13^^,^^18^
Murine MKα3 keratinocytes		^19^
Murine Rac-11P cells		^4^
Phoenix-AMPHO cells	ATCC	CRL-3213
Phoenix-ECO cells	ATCC	CRL-3214
Rat REF-52 cells		RRID:CVCL_6848

**Oligonucleotides**		

*pLKO.1-puro* containing a scrambled sequence and puromycin resistance for selection of positive target cells	TRC Mission library	Cat#C002

**Recombinant DNA**		

*pLV-CMV-mCherry-Vinculin-IRES-Puro,* encoding mCherry-vinculin and puromycin resistance for selection of positive target cells	A kind gift from Dr. J. de Rooij	N/A
*pGFP-Tensin AH2*, encoding GFP-tagged chicken tensin-1	A kind gift from Dr. K. Yamada	^20^
*LZRS-EGFP-KIND1-IRES-zeo, encoding eGFP-tagged kindlin-1 and zeocin resistance for selection of positive target cells*		^21^
*LZRS-ITGB1-IRES-zeo,* encoding human wild-type integrin β1 or mutant β1^Y795A^ and zeocin resistance for selection of positive target cells		^1^^,^^13^
*LZRS-ITGB3-IRES-zeo,* encoding human wild-type integrin β3 or β3 truncation mutants and zeocin resistance for selection of positive target cells		^2^^,^^14^
*LZRS-ITGB4-EGFP-IRES-zeo,* encoding human wild-type integrin β4 subunit and zeocin resistance for selection of positive target cells		^12^
*pHDM-HgpM2 GAG/POL*, encoding the main structural viral proteins and reverse transcriptase	Addgene	Cat#164441
*pRC-CMV-REV1B*, encoding the post-transcriptional regulator Rev1b necessary for efficient synthesis of viral proteins	Addgene	Cat#164443
*pHDMG-G VSV ENV*, coding for the envelope protein of Vesicular Stomatitis Virus	Addgene	Cat#164440
*pHDM-TAT 1B*, encoding the Tat1b protein, which facilitates viral entry into target cells	Addgene	Cat#164442
Imaging software associated with confocal or TIRF microscope (used here: Leica LAS X)	Leica software	https://www.leica-microsystems.com/products/microscope-software/p/leica-las-x-ls/
Leica FRAP wizard	Leica software	https://www.leica-microsystems.com/products/microscope-accessories/p/leica-wf-frap/
ImageJ/Fiji	ImageJ/Fiji software	^22^
MorphoLibJ plugin	ImageJ/Fiji software	^23^
Optional: “*Linear Stack Alignment with SIFT*” plugin for Fiji	ImageJ/Fiji software	^24^
Focal Adhesion Analysis Server (FAAS)	Focal Adhesion Analysis Server (FAAS)	^25^^,^^26^
Macro “*FA Analyzer*”, to be used in Fiji, generated here	GitHub/Zenodo	GitHub: https://github.com/agclark12/FA_analyzer or Zenodo: https://doi.org/10.5281/zenodo.8089114
Microsoft Excel	Microsoft Office software	https://www.microsoft.com/nl-nl/microsoft-365/excel
GraphPad Prism 5.0	GraphPad software	http://www.graphpad.com/scientific-software/prism

**Other**		

Nunc™ Lab-Tek™ II Chambered Borosilicate Coverglasses	Thermo Fisher	Cat#155382
Plasmid DNA purification Mini, Midi, and Maxi kits	Qiagen	Cat#12123, #12143, #12162
Attofluor^TM^ cell chambers	Thermo Fisher Scientific	
T75 and T25 tissue culture flasks	Thermo Fisher Scientific	Cat#169900, Cat#156800
10 cm diameter tissue culture dishes	Thermo Fisher Scientific	Cat#150318
Tissue culture plates, 6 well, 12 well, 24 well	Thermo Fisher Scientific	Cat#140685, Cat#150628, Cat#142485
0.45 μm pore filters	Whatman (GE Healthcare)	Cat#10462100
Circular coverslips, diameter 12–24 mm, no 1.5 (0.16–0.19 mm thickness)	VWR	
Microscope slides	VWR	
Immersion oil for imaging type F	Leica Microsystems	Cat#11513859
Immersion oil for imaging type N	Leica Microsystems	Cat#103699
Parafilm M	Merck	Cat#HS234526C
Tweezers	Leybold	Cat#662029
Bottle top filters	Merck Millipore	S2GVU05RE
Autoclave	N/A	
MoFlo Astrios EQ high speed Cell Sorter	Beckman Coulter	Cat#B25982
Microwave	N/A	
Neon Transfection System	Thermo Fisher Scientific	Cat#MPK5000S
Inverted Zeiss Axiovert 25 microscope with 10× (NA 0.25) and 20× (NA 0.3) objectives, connected to a Zeiss CCD camera (Axiocam MRC) and Zeiss Mr. Grab 1.0 software for image acquisition	Carl Zeiss Microscopy, LLC	N/A
Leica DMI6000 TIRF set-up equipped with multi-position mode, fully motorized Marzhauser stage, and Leica 63× /1.47 oil HCX plan apochromat TIRF objective. Camera used was a Hamamatsu-C9100-02-LNK00.	Leica Microsystems	N/A
Leica DM6000 SP5 confocal microscope with fully motorized Marzhauser stage and 4× HyD and 1× PMT fluorescence detectors and home-made incubation chamber for CO_2_ and temperature-control.	Leica Microsystems	N/A
Zeiss Observer widefield set-up equipped with a plan apochromat 63×/1.40 oil DIC M27 objective, definite focus option, Marzhauser motorized stage, and Hamamatsu Digital Camera, type C10600-10B ORCA-R2. Temperature and CO_2_ were controlled using a home-made incubation chamber.	Carl Zeiss Microscopy, LLC	N/A
Leica DM8 SP8 confocal microscope with fully motorized Marzhauser stage and 4× HyD and 1× PMT fluorescence detectors, Leica 63×/1,40 oil HC plan apochromatic CS objective, and home-made incubation chamber for CO_2_ and temperature-control.	Leica Microsystems	N/A

## Materials and equipment

Primary cells and cell lines that have been used for the experiments described here and our macro “*FA analyzer*” are listed in the above-mentioned key resources table, as well as the necessary reagents, materials, hardware and software for culture, imaging, and analysis. Standard tissue culture disposables are required, as well as a cell culture facility at BSL-1 level, equipped with an incubator and flow cabinet. To express fluorescent proteins, we describe here a transient transfection protocol using a NeonTM Transfection System. For cell culture work involving retroviral and/or lentiviral particles, a BSL-2 facility is necessary, while for working with hazardous reagents a fume hood is required. Materials and reagents for transformation and plasmid isolation, as well as a bacterial culture lab equipped with shaking incubator, are required for the production of constructs encoding shRNAs or (fluorescent) adhesion proteins. Finally, storage space at 4°C, −20°C, and −80°C, as well as a cryostorage facility, is required for maintenance of cells, constructs, culture media and other reagents.

***Alternatives:*** Hoechst and DAPI are both suitable for the labeling of nuclei. Many other antibodies than the ones used here can be used to stain components of cell-matrix adhesions. Similarly, cell-matrix adhesions can be studied in many other adherent cell types than the ones used here. While the transient transfection procedure described here works for certain cell types that are hard to transfect, including keratinocytes and endothelial cells, a wide range of different transfection reagents is available, some of which may work better for particular cell types and are less costly. Furthermore, lentiviral transduction is great to achieve stable expression of fluorescent proteins in cells that are not easily transfected with high efficiency, but is not absolutely necessary since only a few positive cells, which can be easily identified by visual inspection, are sufficient for imaging. Finally, while in this protocol we have used coverslips and Lab-Tek chambers as substrates, a range of tissue culture materials of varying size, shape, and stiffness can be used for imaging. The choice for a specific substrate will depend on the goal of individual experiments, number of conditions, availability of reagents, and the used microscope platform.

• Lennox Broth: Dissolve 20 g in 1 L distilled H_2_O, then autoclave for 15 min at 121°C. Add selection marker (for instance ampicillin) and store at 4°C for up to 4 weeks.

**Table:** DMEM/FCS

Reagent	Final concentration	Amount
DMEM	N/A	440 mL
FCS	10%	50 mL
200 mM L-Glutamine	2 mM	5 mL
D-Glucose	4.5 g/L	2.25 g
100 mM Sodium pyruvate	1 mM	5 mL
Penicillin	100 U/mL	
Streptomycin	100 μg/mL	
**Total**	**N/A**	**500 mL**

**Table:** EGM-2

Reagent	Final concentration	Amount
EBM-2	N/A	500 mL
FCS	2%	10 mL
Growth Medium 2 kit	N/A	N/A
200 mM L-Glutamine	2 mM	5 mL
Penicillin	100 U/mL	5 mL
Streptomycin	100 μg/mL	5 mL
**Total**	**N/A**	**52**5 **mL**

**Table:** Keratinocyte Growth Medium (KGM)

Reagent	Final concentration	Amount
Keratinocyte serum-free medium	N/A	500 mL
Bovine pituitary extract	50 μg/mL	25 mg
EGF	5 ng/mL	2.5 μg
Penicillin	100 U/mL	5 mL
Streptomycin	100 μg/mL	5 mL
**Total**	**N/A**	**5**10 **mL**

***Note:*** The prepared cell culture media can be stored at 2°C–8°C for up to 6 months (DMEM/FCS) or 4 months (EGM-2, KGM).

**Table:** PBS with MgCl_2_/CaCl_2_

Reagent	Final concentration	Amount
PBS	N/A	1 L
MgCl_2_.6H_2_O	0.5 mM	0.10165 g
CaCl_2_.2H_2_O	1 mM	0.147 g
**Total**	**N/A**	**1 L**

***Note:*** To prevent precipitation, the PBS should be swirled while adding MgCl_2_ and CaCl_2_. PBS containing MgCl_2_/CaCl_2_ can be stored at 15°C–30°C for up to 12 months.

**Table:** 2× HBS

Reagent	Final concentration	Amount
Distilled H_2_O	N/A	1 L
HEPES	42 mM	10 g
NaCl	274 mM	16 g
Na_2_HPO_4_	1.4 mM	0.196 g
KCl	10 mM	0.745 g
D-glucose	15 mM	2.7 g
**Total**	**N/A**	**1 L**

***Note:*** Filter-sterilize, aliquot, and store at −20°C for 6 months. To thaw, warm to 20°C and mix by vortexing.

***Note:*** It is essential that the pH is between 7.05 and 7.1.

• 2.5 M CaCl_2_: dissolve 36.75 g CaCl_2_.2H_2_O in 100 mL of distilled H_2_O. Filter sterilize and store aliquots at −20°C.
• 0.1% PBS/gelatin: add 1 g gelatin to 1 L of PBS.

***Note:*** PBS-gelatin should be sterilized before use in tissue culture, for instance using bottle top filters from Millipore (pore size 0.22 μm). PBS-gelatin can be stored for up to 6 months at 4°C.

• 0.1 M glycine: add 7.5 g glycine to 1 L of PBS.

***Note:*** PBS/glycine can be stored for up to 6 months at 4°C.

• PBS/Triton X-100: add 0.4 mL Triton X-100 to 100 mL of PBS.

***Note:*** Triton X-100 is highly viscous. Cut a pipette tip to facilitate pipetting or use a syringe with a plunger. PBS-Triton can be stored for up to 6 months at 4°C.

• 4% paraformaldehyde (PFA): add 40 g PFA to 1 L of PBS.

***Note:*** PFA can be stored at −20°C for up to 12 months.

***Note:*** To make 1 L of PFA solution, first dissolve the powder in 800 mL PBS, stirring constantly. Thereafter, add NaOH drop by drop to clear the solution. Filter the solution, set the pH to 7.0 with HCl, and adjust the volume to 1 L with PBS. For longer storage, aliquot the solution and store at −20°C.

**CRITICAL:** PFA is toxic and carcinogenic and irritates eyes, skin, and airways. Wear a lab coat, gloves, and safety goggles when handling PFA, and work inside a fume hood. Waste should be disposed of in a special container.

***Note:*** As an alternative to freshly preparing PFA, dissolved PFA is also commercially available, for instance as a 16% solution.

• Mowiol: slowly add 2.4 g of Mowiol 4–88 to 6 g of glycerol while stirring, then add 6 mL of distilled H_2_O, mix and leave for 18 h at 20°C. Add 12 mL of 0.2 M Tris/HCl (pH 8.5) and heat to 50°C for 10 min while mixing occasionally. When the Mowiol has dissolved, clarify the solution by centrifugation at 5000 × *g* for 15 min. Add up to 2.5% DABCO to reduce fluorescence fading.

***Note:*** Aliquots can be stored at −20°C for up to 12 months.

## Step-by-step method details

### Staining of cell-matrix adhesions for immunofluorescence imaging

**Timing: 1–2 days**

This method describes how to prepare cells on coverslips and stain individual components of cell-matrix adhesions in fixed cells. Work in the flow hood to keep cells and materials sterile. While we grow cells here on coverslips, other formats including glass-bottom imaging plates or Lab-Tek chambers can be used, depending on individual requirements.

1. Coat coverslips with the desired ECM protein as described in step 3 of prepare buffers, cell culture media, and substrates.
2. Trypsinize cells, seed the appropriate cell density in culture medium on coated coverslips in a 24-well plate, and place in the incubator at 37°C and 5% CO_2_.

***Note:*** The time required depends on the cell type, matrix used, the type of adhesion complex to be visualized and the purpose of the experiment. While FA formation during initial cell adhesion and cell spreading can be visible as early as 10 min after seeding, other complexes such as FBs may appear later in cell cultures where there is also abundant FN deposition. HDs are usually best visualized by first letting cells adhere and spread in culture medium, and then incubate the cells in high calcium for 18 h (to stimulate HD assembly) prior to fixation.

***Note:*** 12-mm diameter circular coverslips are recommended for a 24-well plate.

***Note:*** (Transfection or transduction with) different constructs may affect proliferation in different ways. By seeding multiple coverslips per condition in different densities, coverslips with similar confluency can be selected at the time of the experiment.

3. Perform any required experiment (i.e., drug treatment, cytokine or growth factor addition, etc).
4. Aspirate the medium and wash once with PBS containing MgCl_2_/CaCl_2_ to remove medium and/or serum components.

***Note:*** MgCl_2_ and CaCl_2_ are required to maintain integrin- and cadherin-dependent cell adhesion complexes, respectively.

5. Fix with 500 μL of 4% PFA for 15 min at 20°C.

**Pause point:** Cells fixed with PFA can be stored at 4°C for up to a week. Seal the plate with parafilm to avoid evaporation.

6. Wash 2 times with PBS.
7. Permeabilize with 500 μL of 0.5% Triton X-100 in PBS for 5 min at 4°C.
8. Wash Triton away with 2 washes in PBS.

***Note:*** The use of MgCl_2_ and CaCl_2_ in PBS is no longer required after fixation. However, some antibodies may require calcium for binding.

9. Block non-specific binding with 0.1 M glycine in PBS for 2 h at 20°C.

**CRITICAL:** Glycine blocks reactivity of free aldehyde groups that can bind antibodies, and thereby reduces non-specific (background) staining. Other strategies to block non-specific binding include the use of bovine serum albumin fraction V, serum, or fish skin gelatin (troubleshooting 4).

10. Wash coverslips 3 times with PBS containing 0.2% gelatin.
11. Dilute primary antibodies in PBS with 0.2% gelatin.
12. Place a piece of clean parafilm on the bench and pipet drops of antibody solution, evenly spaced, on it. For a 12-mm coverslip, 30 μL should suffice.

**Figure 3:**
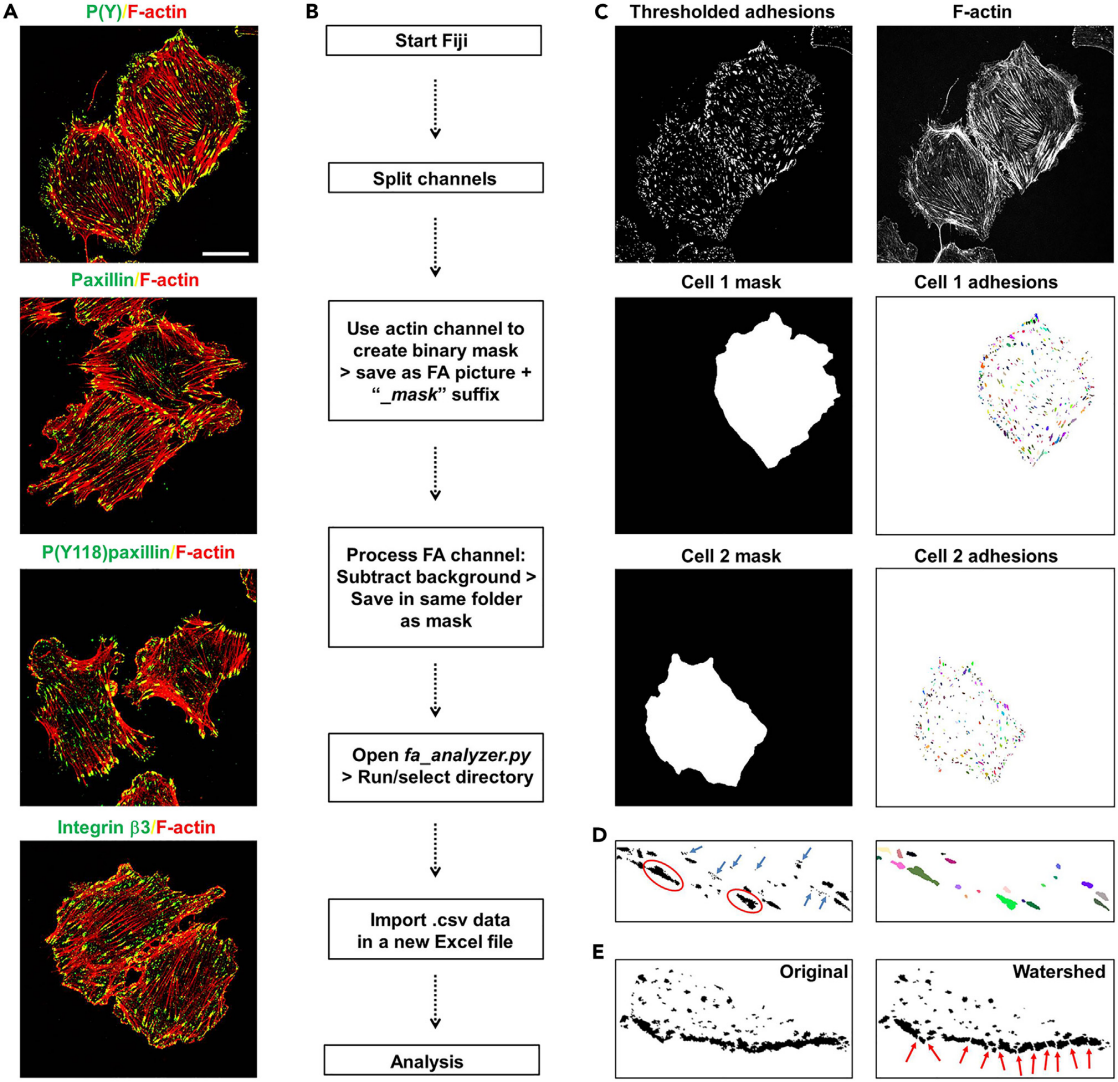
Workflow for the quantitative analysis of FAs in fixed cells using the *FA analyzer* macro (A) Confocal images (obtained on a Leica SP8 system as described in confocal imaging of cell-matrix adhesions in fixed cells) showing the visualization of FAs in GEβ3 cells using antibodies against integrin β3, paxillin, phospho(Y118)paxillin, and phosphotyrosines (PY; *green*), co-stained with F-actin (*red*). Bar, 20 μm. (B) Workflow for the analysis of FAs using the *FA analyzer* macro generated here. (C) The macro segments FAs on confocal images by thresholding and watershed, identifies individual FAs within the masked area (generated on the actin channel), and performs quantitative assessment of FA properties. (D) Examples of unrealistically small FAs (left, *blue arrows*) which can be excluded from analysis based on size (right). Similarly, clusters of adhesions that are not properly segmented (*red ovals*) and are therefore arbitrarily counted as 1 large FA, could also be excluded. (E) Example of the effect of watershed (right, *red arrows*) on FA segmentation (compare to original image left).

***Note:*** This step is to reduce the amount of antibody used for labeling. Alternatively, staining can be performed by incubation of the coverslips in the well plate but this requires much larger volumes.

***Note:*** Several different antibodies can be used to visualize FAs that will result in similar staining, including antibodies directed against vinculin, paxillin, focal adhesion kinase (FAK), phosphorylated paxillin or FAK, or phosphotyrosines in general (Figure 3A). The choice of which marker is best will depend on cell type and individual demands.

13. Put 1 coverslip (with cells down) on each drop of antibody solution.

***Note:*** Handle coverslips using tweezers with a pointed tip. To grab coverslips with tweezers, they can be lifted easily with a syringe needle that has been pressed onto a hard surface to create a lightly-bended tip.

***Note:*** Remove superfluous PBS on the coverslip by gently holding it on its side against a tissue.

14. Incubate for 45–60 min at 20°C.
15. Put coverslips back in PBS with 0.2% gelatin in the 12-wells plate with the cells facing up.
16. Wash cells 3 times with PBS containing 0.2% gelatin for 10 min.

**CRITICAL:** When aspirating medium and replenishing, be careful to avoid cells drying out during the staining procedure.

17. Dilute secondary antibodies in PBS with 0.2% gelatin and pipet drops on parafilm as in step 12. Phalloidin and Hoechst can also be included in this step.
18. Put coverslips on antibody solutions as in step 13.
19. Incubate for 30–60 min at 20°C in the dark.

**CRITICAL:** We use phalloidin to visualize actin filaments (F-actin) and Hoechst to image the nuclei. Both stains are very bright and stain quickly (Hoechst stains already within 5 min). In general, it is recommended to first determine the best conditions for labeling of phalloidin and antibodies (ie concentration, incubation times). Some antibodies stain better on MeOH-fixed cells than on cells fixed with PFA (troubleshooting 4).

**CRITICAL:** Phalloidin is dissolved in DMSO. Wear protective clothing, gloves, and eye/face protection when handling DMSO or the DMSO dye solution. DMSO easily penetrates the skin and facilitates the absorption of organic molecules into tissues. Dispose of waste and materials in compliance with the appropriate local regulations.

20. Transfer coverslips again to plate as in step 15.
21. Wash coverslips 3 times with PBS containing 0.2% gelatin.
22. Wash coverslips twice with PBS.
23. Mount coverslips with cells face-down in 10% Mowiol on object slide. For 12-mm coverslips, 10–20 μL of Mowiol is sufficient.

***Note:*** Mowiol is viscous. To facilitate pipetting, use pipette tips with cut opening.

***Note:*** 5% Dabco 33-LV (an anti-fade agent) can be added to the Mowiol before mounting.

***Note:*** Very gently press the coverslip down to bring it close to the object slide.

24. Let Mowiol harden for at least 16 h at RT.
25. Carefully clean the non-cell-side of the mounted coverslip with a tissue with EtOH, then store coverslips at 4°C or −20°C.

***Note:*** Sealing coverslips with clear nail polish along their edge will prevent dehydration.

**Pause point:** Coverslips mounted in Mowiol can be stored for weeks at 4°C or even months at −20°C.

***Note:*** Instead of Mowiol, also commercial mounting media can be used, such as Vectashield. Some of these contain DAPI to stain nuclei during embedding, or ‘anti-fade agents’ to protect fluorescent dyes from fading due to photobleaching.

### Confocal imaging of cell-matrix adhesions in fixed cells

**Timing: 1–8 h**

This method describes how to image cell-matrix adhesions in cells on coverslips, using an inverted confocal laser scanning microscope such as the Leica SP8 system used here.

26. Select the 63× objective (used here: Leica 63×/1,40 oil HC plan apochromatic CS objective).
27. Apply immersion oil type F on coverslip and place upside down (facing the objective) in the microscope stage insert.
28. Select the appropriate laser lines. We used excitation at 405 nm for Hoechst, 488 nm for FITC- or AlexaFluor 488, 561/594 nm for Texas Red, and 633/647 nm for Cy5).
29. Set laser/gain and other confocal settings. Here we acquired images with: image dimensions 2048 × 2048 pixels, scan speed 200 Hz, bidirectional scanning, and performing sequential scanning between channels (to avoid crosstalk between fluorophores).
30. Select the basal focal plane to focus on cell-matrix adhesions and optimize laser and gain settings.
31. To reduce noise and enhance specific signal, perform line or frame averaging.

***Note:*** When comparing different conditions, capture images with the same confocal settings for all conditions (first optimize settings on the control condition).

***Note:*** The settings mentioned here will result in high-resolution images. For the imaging of large numbers of cells/conditions, scanning time can be reduced using resonant scanning or by limiting the number of averaging steps. In addition, lower image dimensions (e.g., 1024 × 1024 or 512 × 512) will reduce the time required for imaging. Finally, simultaneous acquisition can be used, rather than sequential scanning. All of these steps will result in lower resolution but may still be sufficient for proper visualization and quantification.

***Note:*** Because of cell-to-cell heterogeneity and variability in FA number and size, it is advised to image and analyze multiple cells (at least 10) per condition.

### Automated analysis of cell-matrix adhesions in fixed cells

**Timing: 5 min–2 h**

**Figure 4:**
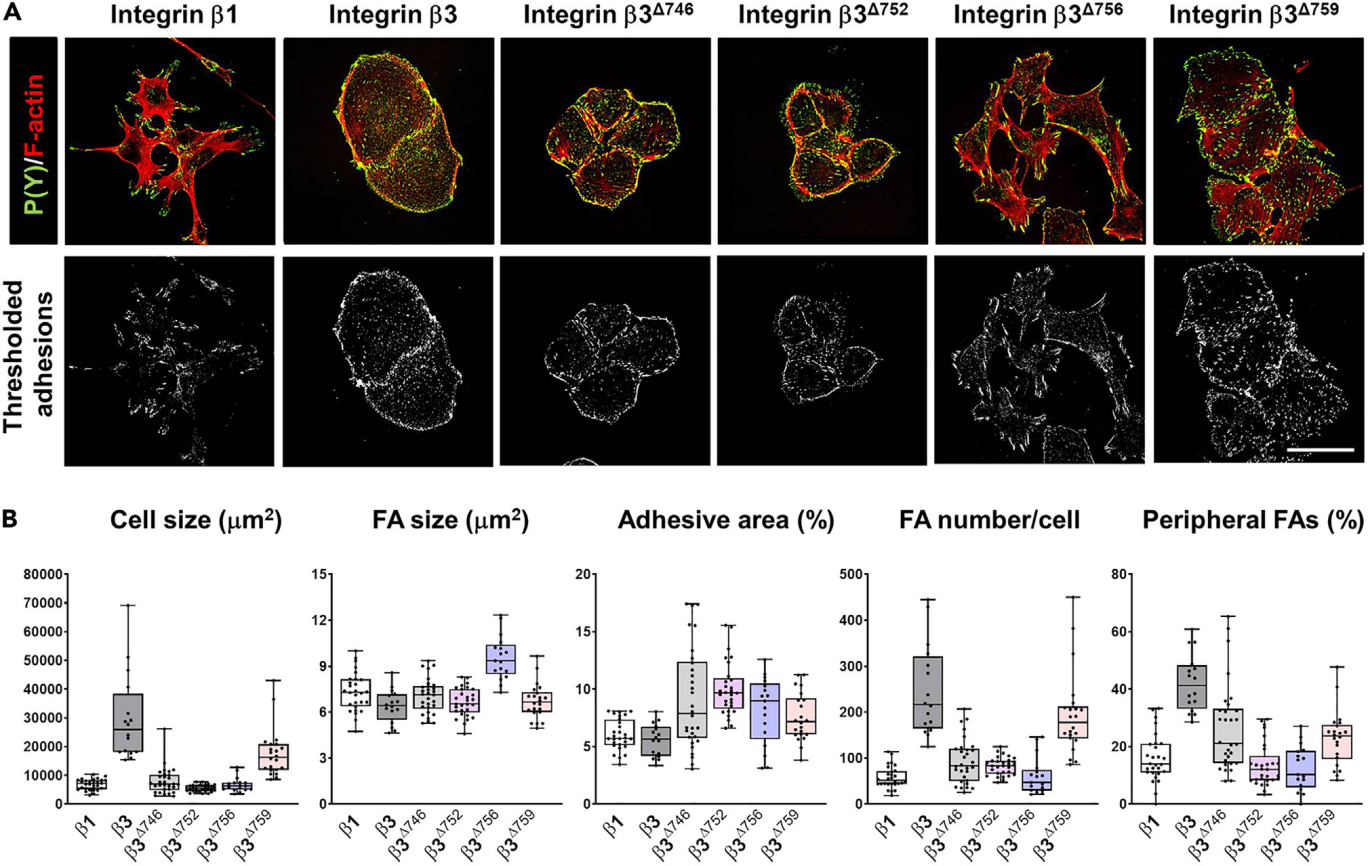
Examples of quantitative FA analysis in fixed cells (A) Confocal images of GE11 cells expressing integrin β1, integrin β3, or the indicated β3 mutants, showing FAs (*green*) and F-actin (*red*), and thresholding of the FA channel of these images. Images were collected as described in confocal imaging of cell-matrix adhesions in fixed cells. Note that different integrins (β1 versus β3) or integrin mutants can have very different effects on cell morphology, as well as on the formation, number, size and appearance of cell-matrix adhesions. Bar, 40 μm. (B) Box and whisker plots showing quantifications of FA size and number, cell size, adhesive area, and fraction of peripheral adhesions using the *FA analyzer* macro on the cell lines shown in (A). Data are averages +/-SD per cell, derived from 15-29 cells per condition. Bars, 40 μm.

This method makes use of the macro “*FA Analyzer*” developed by us, which is available from GitHub:https://github.com/agclark12/FA_analyzer. The package contains a single Python/Jython script that can be opened and run directly in Fiji/ImageJ. Here we exemplify the use of this macro on FA/F-actin confocal images. The macro will identify and analyze individual FAs on pre-processed images as indicated below (Figure 3B). Thresholding is a common step to segment adhesions. The macro uses a thresholding algorithm to overcome the need for manual input of values for thresholding. Moreover, the macro includes a watershed step for further segmentation of individual adhesions (Figures 3C, 3D and 4A). The resulting data can be used to extract FA properties from single cells or combined to look at average properties in cell populations (Figure 4B). The required time for analysis will depend on the number of images, binning options, and the computer used.

32. Split the merged image containing FA and F-actin channels into single channel images using *Image* > *Color* > *Split channels.*
33. Perform background subtraction using *Process* > *Subtract background.*
34. Generate a mask on the F-actin channel with the segmented cell to analyze, by drawing a line around the cell boundary with the *Freehand Selection Tool* (Figures 3B and 3C).

***Note:*** As long as the mask image is an 8-bit binary image (all pixels have a value of either 0 or 255), masks can also be generated by different means, for example using an automated segmentation in a previous analysis step.

35. Save the selection with *Edit* > *Selection* > *Add To Manager.*
36. Create a new image using *File* > *New* > *Image* (Type: 8-bit, Fill with: Black, Width/Height: same as FA image) > *OK.*
37. Select the saved cell boundary from the region of interest (ROI) manager.
38. Ensure that the foreground color is white using the *Color Picker Tool.*
39. Fill the region in the new image using *Edit* > *Fill.*
40. Save using the same name as the corresponding FA image, but with "*_mask*" appended to the end (for "*cell2.tif*", the mask image should be named "*cell2_mask.*tif").

***Note:*** The naming convention must remain (always use the "*_mask*" suffix). Changing the naming convention or file type (in case another format than Tiff images is used), can be done in *fa_analyzer.py* in the main function at the bottom of the script.

41. Install the necessary files and folder structure.
  a. To run the *FA Analyzer*, first install the MorphoLibJ plugin.^23^ The easiest way to do this is to subscribe to the IJPB-Plugins site via the Fiji update sites.
  b. Copy the .txt file to the macro folder of ImageJ/Fiji (standard C:∖Program Files∖ImageJ∖macros).
  c. Before running the script, on Fiji, go to *Process* > *Binary* > *Options* and ensure the box “*Black Background*” is ticked.
42. Run macro.
  a. Open *fa_analyzer.py* in Fiji.

***Note:*** This will bring up an editor window showing the script. Click *Run*. Upon running the script, you will be prompted to select a directory. This directory should contain all of the FA images to be analyzed (for example, all images from a particular condition/experiment). The FA image should be single channel, 8- or 16-bit, any image size is fine.

***Note:*** The script will automatically create new directories for storing data, which will be named according to the FA images. Within these directories, there will be .csv files saved with data for each individual FA (“*FA data*”) and data related to FAs at the whole-cell level ("*image data*"). Furthermore, images with all identified FAs (indicated by distinct colors) are also included (Figure 3C).

***Note:*** The FA data include the following parameters:

 *fa_area_px* : area of the FA (in pixels)

 *perim_px* : perimeter of the FA (in pixels)

 *x0_px* : x-coordinate of the FA centroid (in pixels)

 *y0_px* : y-coordinate of the FA centroid (in pixels)

 *orientation_deg* : orientation angle of the FA (from −90 to 90 degrees)

 *ar* : aspect ratio of the FA (length of major axis/minor axis)

 *sf* : shape factor of the FA (another measure of elongation, defined as sf = perimeterˆ2/area)

 *dist_px* : minimal distance from the FA centroid to the cell border (in pixels)

 *angle_deg* : angle of the FA centroid with respect to the cell centroid (in degrees)

 *rel_orientation_deg* : relative orientation of the FA orientation with respect to the angle to the centroid (0 degrees means orientation is parallel with the angle to the centroid, 90 degrees means the orientation is perpendicular with the angle to the centroid)

***Note:*** The whole-cell level ("*image*") data include the following additional parameters:

 *mask_area_px* : area of the cell mask (in pixels)

 *no_fas* : total number of FAs within the cell mask area

 *area_fraction* : fraction of total FA area to cell area

 *frac_periph_fas* : fraction of "peripheral" FAs

***Note:*** As an alternative to opening and running the script from the editor in ImageJ/Fiji, you can also install it as a plugin by copying “*fa_analyzer.py*” to the ImageJ plugins folder. Then restart ImageJ and you will be able to find and run FA analyzer directly from the “*Plugins*” menu.

***Note:*** By default, the distance of "peripheral" FAs to the cell border is less than 10% of the maximum distance from the border to the center of the cell; this 10% fraction is defined in the main function at the end of the script and can be changed, but it should be kept consistent for all images that are to be compared.

***Note:*** The means and standard deviations (std) of most of the FA parameters are also stored in the image data.csv file. For convenience, all of the FA and image data are collected together in .csv files that are saved to the directory containing the images.

***Note:*** Area and perimeter data are always given in pixels. This avoids errors due to inconsistencies in images that already have pixel sizes defined in the image metadata. It is recommended to convert such values to real physical values (microns, e.g.). As this conversion will be different for individual images that were acquired with different imaging parameters (zoom/objectives, e.g.), it is best to be consistent with imaging parameters for a given set of images that will be analyzed together.

43. Analyze data.
  a. Close ImageJ and open the .csv files.
  b. Analyze data.

***Note:*** To exclude unrealistically small FAs, the cut-off for adhesion size can be adjusted in the sheet (Figure 3D). A similar approach can be adopted to exclude FAs that are unrealistically large, or clustered FAs that are not properly segmented and therefore appear as 1 large FA rather than several small ones (Figure 3D). Focal adhesion size is on average between 1 and 10 μm in most cell types.

***Note:*** Graphs can be created directly in Excel or using GraphPad Prism. The latter also allows direct statistical analysis. Start from data table or graph → *Analyze* → choose the appropriate method of statistical analysis, depending on experimental set-up and number of conditions.

***Note:*** It is recommended to use images with a maximal pixel size of 0.15–0.3 μm/pixel to allow for accurate detection and separation of individual FAs. In addition, sufficiently high signal-to-noise must be achieved in order to detect FAs from the background signal. Improvement of signal-to-noise ratio prior to analyzing the FAs is performed by background subtraction in Fiji. If the background signal is still too high, consider adjusting the staining conditions (troubleshooting 4).

***Note:*** Segmentation of individual FAs may be difficult or even impossible in cases where adhesions form large clusters. Watershed is performed by the macro to perform FA segmentation but may also induce artefacts (see Figure 3E) (troubleshooting 5). In cases where FAs seem continuous and segmentation is problematic, the adhesive area may be more informative than the number of adhesions (see for example the adhesions formed by the β3^Δ756^ mutant in Figure 4).

### Live imaging of FAs using TIRF microscopy

**Timing: 30 min–8 h**

**Figure 5:**
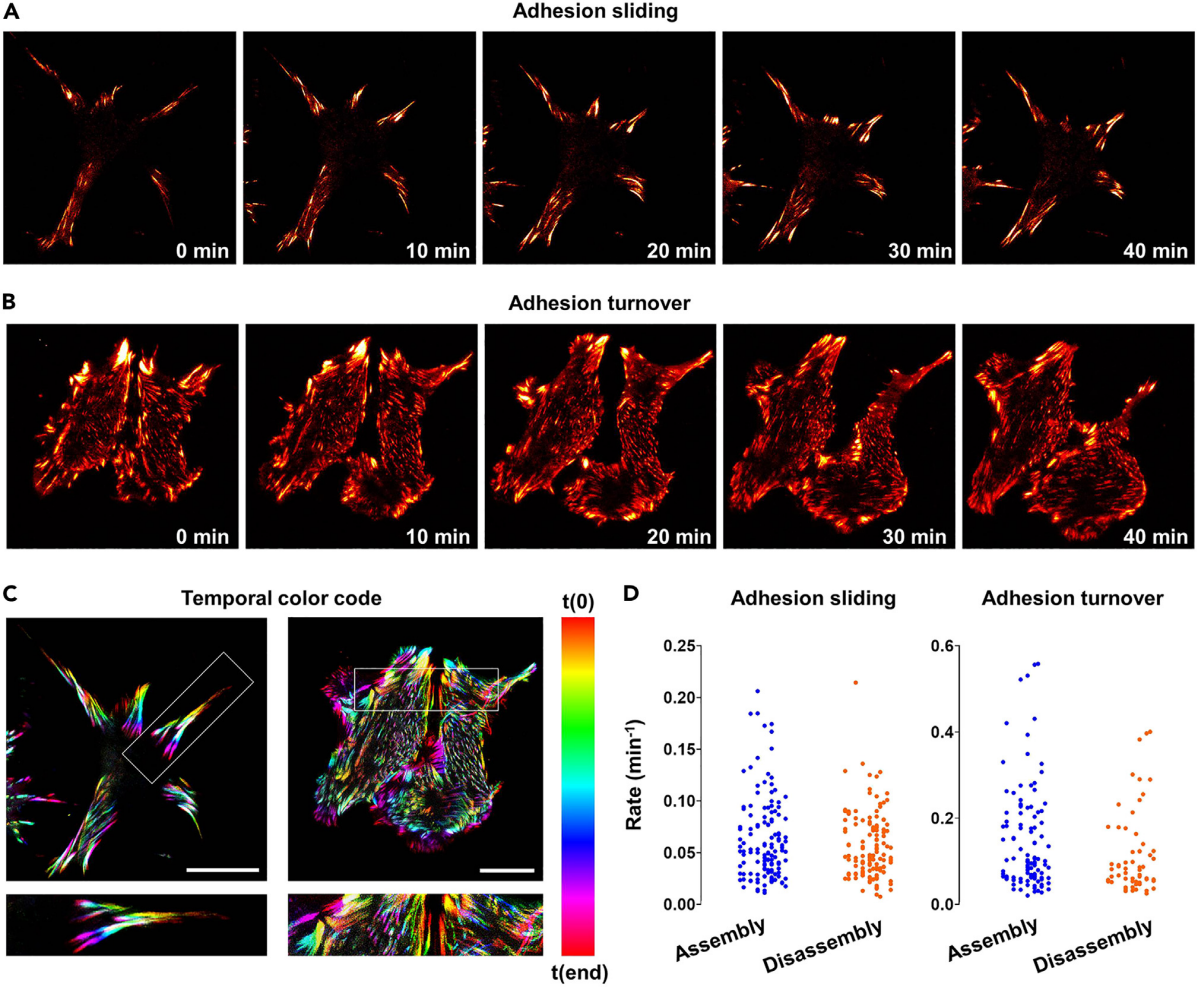
Live imaging and analysis of dynamic FA behavior using TIRF microscopy (A and B) Still images from time-lapse series of GEβ1 (A) and GEβ1^Y795A^ (B) cells expressing mCherry-vinculin and imaged using a Leica TIRF set-up as described in live imaging of FAs using TIRF microscopy. Used LUT, *RedHot*. Images in (A) show mainly persistent FAs that migrate toward the cell center during cell contraction (an example of sliding adhesions), while images in (B) show mainly rapid FA assembly and disassembly (adhesion turnover). (C) Color-coded time projections of Methods video S1 and S2 were generated using LUT *Spectrum*. Bars, 25 μm. (D) Quantification of FA assembly and disassembly rates using the *FAAS*. Each point represents an individual cell. See also Methods video S1 and S2.

This method describes how to collect images of the dynamic behavior of FAs by time-lapse TIRF imaging, using mCherry-vinculin in GE11 cells expressing wild-type or mutant integrin β1 (Methods video S1 and S2; Figure 5). Imaging was performed on a Leica DMI6000 set-up, using a 63× NA 1.47 plan apochromatic TIRF objective and equipped with multi-position mode, fully motorized Marzhauser stage, and Hamamatsu-C9100-02-LNK00 camera.^1^^,^^21^ A custom-made enclosure for temperature control and CO_2_ was used. While this example illustrates general procedures, it can of course be applied to other cell types and/or fluorescent proteins.

44. Transduce GE11 cells expressing wild-type integrin β1 or mutant β1^Y795A^ with a construct encoding mCherry-vinculin, as described in lentiviral transduction for stable expression of fluorescent adhesion proteins or shRNAs.
45. Seed cells on large circular coverslips (24 mm diameter) that have been coated with FN (or another appropriate ECM protein) as described in prepare buffers, cell culture media, and substrates, and are maintained in 6 well plates.
46. Replace the medium approximately 1 h before starting to set up the microscope.

***Note:*** To increase cell motility and dynamic adhesion behavior, cells can be serum-starved prior to the experiment for 1–4 h, then re-stimulated with fresh medium containing serum shortly before onset of the experiment.

47. Apply immersion oil type N on the 63× objective. Secure a 24 mm diameter coverslip with cells into the coverslip chamber of the microscope.

***Note:*** The microscope should be prewarmed and atmospherically stabilized at 37°C and 5% CO_2_.

***Note:*** While here we have used a system where individual circular coverslips are secured into a circular coverslip chamber, other TIRF systems have different options to mount cells, for instance in glass bottom dishes.

48. Select the appropriate laser lines and settings (intensity, time interval, etc). Appropriate time intervals will depend on the purpose of the experiment, the cell type, the expression levels of the protein under investigation, and the type of adhesion structure to be analyzed.

***Note:*** Here we used excitation wavelength of 561 nm for mCherry, exposure time 283 ms, laser intensity 18%, interval 1 min, total imaging 45 min, angle 68°, penetration depth 90 nm.

49. For multi-position imaging, select xyz positions on the coverslip and save to positions list. Attempt to image several different fields per condition.
50. Ensure all positions are still in focus. Use of the hardware autofocus control AFC may be necessary (troubleshooting 6). This option is called Definite Focus on Zeiss microscopes and Perfect Focus on Nikon machines.
51. Start the experiment.

***Note:*** BSL-2 imaging facilities are required in case imaging is performed shortly after transduction.

***Note:*** The normal behavior of adhesion complexes, as well as proper capture of their dynamics, is influenced by many different factors (troubleshooting 7).

***Note:*** Risks of live-cell fluorescent imaging include signal bleaching and phototoxicity, which can be minimized with a number of approaches (troubleshooting 8).

Methods video S1. Sliding FAs, related to steps 44-51 of “live imaging of FAs using TIRF microscopy” Time-lapse movie of GEβ1 cells expressing mCherry-vinculin. Images were acquired on a Leica TIRF set-up. Magnification 63× (oil), interval 1 min, total time 45 min. See also Figure 5.

Methods video S2. Rapid FA turnover, related to steps 44-51 of “live imaging of FAs using TIRF microscopy” Time-lapse movie of GEβ1^Y795A^ cells expressing mCherry-vinculin. Images were acquired on a Leica TIRF set-up. Magnification 63× (oil), interval 1 min, total time 45 min. See also Figure 5.

### Live imaging of FBs using widefield microscopy

**Timing: 30–90 min**

**Figure 6:**
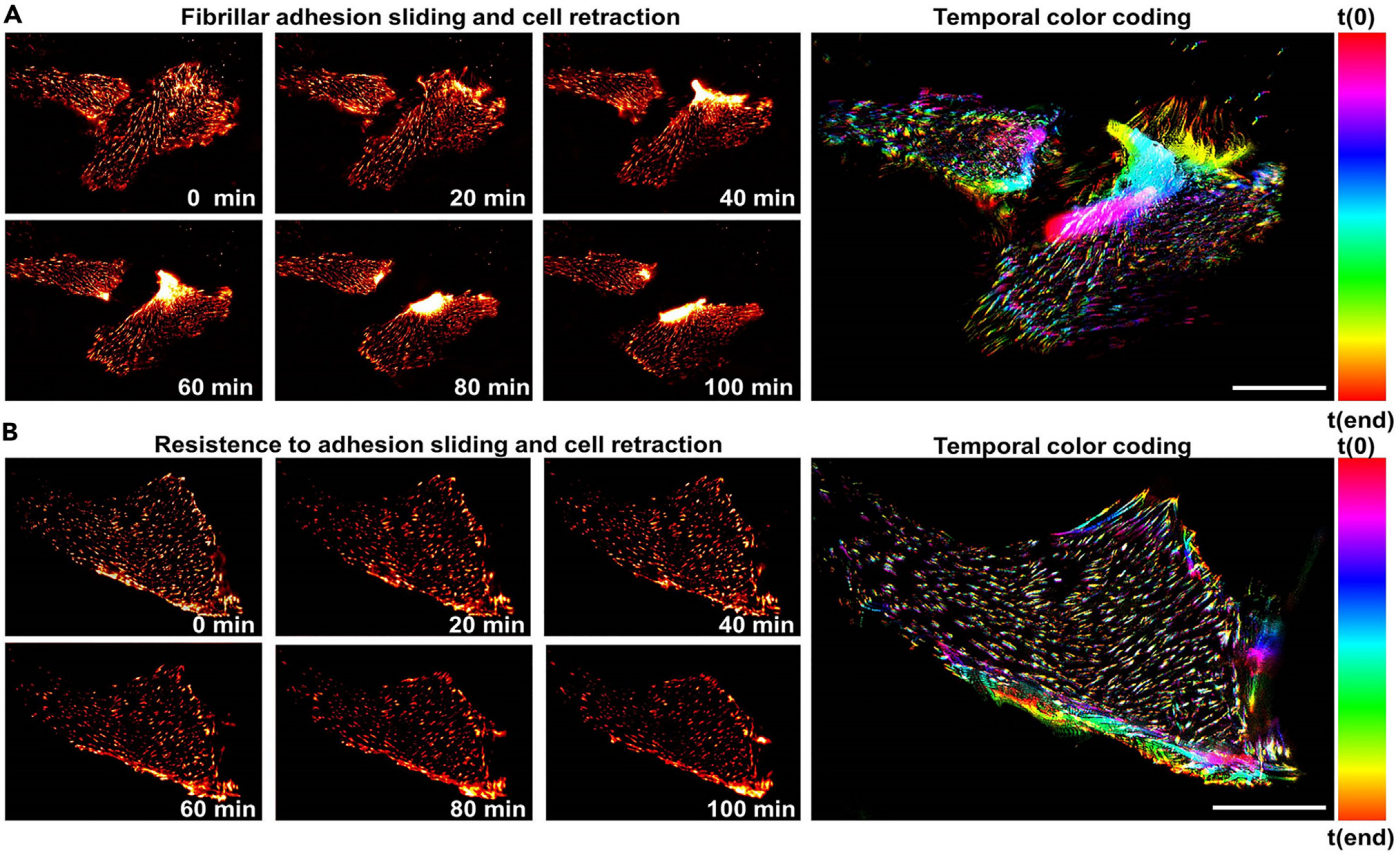
Live imaging of FBs using widefield microscopy (A and B) Still images from time-lapse series of HUVECs in monolayer, transiently transfected with a construct encoding GFP-tensin-1 and stimulated with thrombin (1 U/mL). Used LUT, *RedHot*. Movies were acquired using a Zeiss Observer widefield set-up as described in live imaging of FBs using widefield microscopy. Images on the right and magnification of boxed regions are color-coded time projections (using LUT *Spectrum*). Note the strong polarized accumulation of tensin-1 preceding cell retraction in (A). Bar, 25 μm. See also Methods video S3.

In this method, we describe how to collect images of the dynamic behavior of FBs after thrombin stimulation by widefield imaging, using GFP-tensin-1 in HUVECs with stable knockdown of Arg/Abl2 expression (Methods video S3; Figure 6).^3^ Here we used a Zeiss Axiovert Observer Z1 system equipped with a plan apochromat 63×/1.40 oil DIC M27 objective with a Metal Halide illumination system and a Hamamatsu digital camera type C10600-10B ORCA-R2, but other set-ups can also be used. Similarly, this approach can be applied to investigate the function of other proteins and in other cell types.

52. Transduce HUVECs with a scrambled sequence or shRNAs to suppress expression of a protein of interest (in this case Arg/Abl2), as described in lentiviral transduction for stable expression of fluorescent adhesion proteins or shRNAs.

***Note:*** Be sure to transduce sufficient cells to be able to yield 3–5 × 10^5^ cells per condition.

53. 24 h after transduction, replace medium with fresh culture medium containing 1 μg/mL puromycin.
54. 48 h after transduction, collect the cells and microporate them with GFP-tensin-1, as described in transient expression of fluorescent proteins in cells with stable gene suppression.
55. Seed cells in medium containing puromycin in a 6 well plate.
56. Coat an 8-well Lab-Tek chamber with FN as described in prepare buffers, cell culture media, and substrates.
57. 72 h after transduction/24 h after microporation, harvest cells from a confluent well in a final volume of 1 mL culture medium with puromycin.
58. Distribute the cells in different densities over 4 Lab-Tek chamber wells by adding 100 μL, 200 μL, 300 μL, 400 μL of cell suspension per well.
59. Fill up to 500 μL with cell culture medium by adding 400 μL, 300 μL, 200 μL, 100 μL per well, respectively.

***Note:*** Seeding of different cell densities allows for correction of differences in growth rate which may occur as a result of transduction and/or microporation with different constructs. Look at wells under an inverted tissue culture microscope at low magnification (such as a Zeiss Axiovert 25 microscope at 10× (NA 0.25) or 20× (NA 0.3) magnification, as described in cell culture and passage) and select those that have similar cell density for the experiment.

60. 96 h after transduction/48 h after microporation, replace medium with 400 μL of fresh medium without puromycin. Allow cells to stabilize for at least 2 h before adding thrombin.
61. Apply immersion oil type N on the 63× objective. Secure the Lab-Tek chamber in the correct table insert. The microscope should be prewarmed and atmospherically stabilized at 37°C and 5% CO_2_.
62. Select the appropriate settings for imaging, which will depend on the microscope used, the purpose of the experiment, the cell type, and the type of adhesion structure to be analyzed.

***Note:*** Here we used the following settings: excitation wavelength, 495 nm; filter excitation wavelength, 450–490 nm; emission wavelength, 519 nm; filter emission wavelength, 500–550 nm; light source intensity, 73.95%; , exposure time, 600 ms; depth of focus: 0.80 μm).

***Note:*** Selecting a single positive cell surrounded by negative cells for imaging, makes downstream analysis with FAAS easier.

***Note:*** Live imaging of adhesions can also be combined with imaging of the actin cytoskeleton, for which several approaches exist to monitor cytoskeletal changes in migrating cells.^27^ For example, Lifeact can be used^28^ but this requires another transfection or transduction. Alternatively, cells can be live-labeled with probes such as those from Spirochrome, which are cell-permeable, photostable and fluorogenic, enabling live-labeling and imaging with low background and limited phototoxicity.^29^ In HUVECs, efficient labeling is achieved by incubating the cells with these probes 2 h prior to the experiment, and keeping them in the medium during imaging for up to 24 h.^30^

63. For multi-position imaging, select xy positions and save to positions list. Attempt to image several different fields per condition.

***Note:*** Use of the Definite Focus option will be necessary (troubleshooting 6).

64. Start the experiment.
65. 5 min after starting the experiment, very carefully lift the lid of the Lab-Tek chamber. Check live whether the correct positions are still being imaged.
66. Add 100 μL of 2.5 U/mL thrombin to a well already containing 400 μL of medium, for a final concentration of 0.5 U/mL thrombin.
67. Check live whether the correct positions are still being imaged. Very carefully place the lid back.

***Note:*** Bending a fine gel-loading tip will facilitate pipetting into the Lab-Tek chamber wells while the sample is on the microscope stage.

***Note:*** When comparing different conditions, capture images with the same settings for all conditions (first optimize settings on the control condition).

***Note:*** BSL-2 imaging facilities are required in case imaging is performed shortly after transduction.

***Note:*** The normal behavior of adhesion complexes, as well as proper capturing of their dynamics, is influenced by many different factors (troubleshooting 7).

***Note:*** Risks of live-cell fluorescent imaging include signal bleaching and phototoxicity, which can be minimized with a number of approaches (troubleshooting 8).

Methods video S3. Dynamics of FBs, related to steps 52-67 of “live imaging of FBs using widefield microscopy” Time-lapse movies of HUVECs in monolayer, expressing GFP-tensin-1 and stimulated with thrombin (1 U/mL). Images were acquired using a Zeiss Observer widefield set-up. Note that the cells on the left show sliding of FBs and cell retraction in response to thrombin, while the cell on the right is resistant to this effect. Magnification 40×, interval 1 min, total time 60 min. See also Figure 6.

### Quantitative analysis of cell-matrix adhesion dynamics in live cells

**Timing: 1–2 days**

After acquisition of images by one of the live-cell imaging approaches described above, image stacks can be uploaded to FAAS for analysis of adhesion dynamics.^25^^,^^26^

***Note:*** This software segments all individual adhesions in each frame and tracks their fate through consecutive frames, thereby enabling calculation of adhesion lifetime and assembly and disassembly rates. As for the analysis of adhesion complexes in fixed cells, it is essential that the complexes under investigation appear as discrete structures that can be recognized as individual entities and thus properly segmented by the software. Therefore this type of analysis is not suitable for large structures such as HDs, while it can be applied in many instances to FAs and/or FBs.

***Note:*** It is crucial that the software recognizes the same adhesion in consecutive frames. Too large time intervals between images, as well as differences in quality between subsequent images may cause the software to lose track of particular adhesions.

***Note:*** FAs and FBs can display distinct modes of dynamic behavior. Some adhesions have a long lifetime and thus low turnover rates, and can move along the ligand, largely following cellular morphological changes (‘sliding adhesions’). In contrast, adhesion dynamics can be characterized mainly by rapid disassembly and *de novo* re-assembly, leading to shorter lifetime and high turnover rates (see Methods video S1, S2, Figures 5 and 6 for examples).

***Note:*** The quality of the input data is crucial for proper analysis. Therefore, care should be taken to make sure movies are in-focus, with low background signal, and minimal photobleaching (troubleshooting 6, 7, and 8).

### Live imaging of HDs using TIRF microscopy

**Timing: 5–12 h**

**Figure 7:**
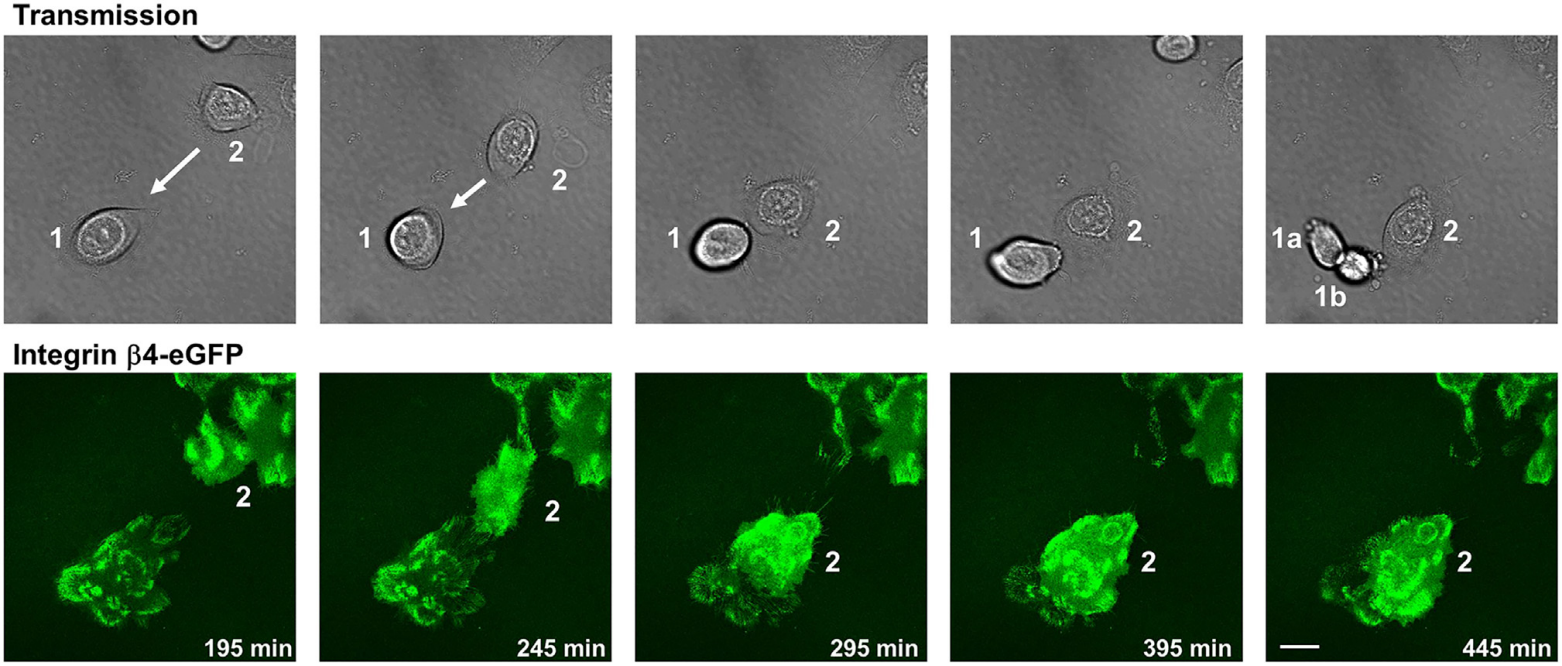
Live-cell imaging of dynamic HD behavior using TIRF microscopy Still images from a time-lapse series of PA-JEB keratinocytes stably transduced with β4-eGFP, and treated with 50 ng/mL EGF to stimulate cell proliferation and migration. Cells were imaged by TIRF microscopy as described in live imaging of HDs using TIRF microscopy. Upper panels, DIC channel; lower panels, GFP channel. Keratinocytes stably adhere via HDs to laminin-332 that they have deposited themselves. To migrate, keratinocytes need to disassemble their HDs. HDs are also disassembled during cell division, although some α6β4 remains present in retraction fibers to keep the round, mitotic cells anchored to the substratum. Note that the patches of laminin-332 that are no longer occupied by integrin α6β4 of the mitotic cell (cell #1) are seized by the migrating cell (#2), thus hindering post-mitotic cell spreading of the two daughter cells (#1a and #1b). Bar, 80 μm. See also Methods video S4.

In addition to the imaging of fluorescent adhesion complex components, fluorescent integrins can also be used for live-cell imaging. Fluorescent tags at integrin cytoplasmic tails permit imaging of integrin dynamics and FA behavior,^31^ while ecto-tagging allows to follow the dynamics of integrins through the endolysosomal system.^32^ Here we describe live imaging of dynamic HD behavior after EGF stimulation in PA-JEB keratinocytes that express integrin β4 with an eGFP tag at the cytoplasmic tail, using TIRF microscopy (Methods video S4; Figure 7). A similar approach can be applied to look at the dynamics of other integrins, as well as in other cell types.

68. Place large circular coverslips (24 mm diameter) into the wells of a 6-well plate.
69. Collect and seed PA-JEB cells expressing β4-eGFP on the coverslips in KGM and incubate the cells for at least one day in a humidified incubator at 37°C, 5% CO_2_.
70. Replace the KGM with DMEM/FCS and incubate for an additional day in a humidified incubator at 37°C, 5% CO_2_.
71. Place the coverslips with cells in an Attofluor™ Cell Chamber (Thermo Fisher Scientific) for live-cell imaging and mount in a 35 mm diameter stage holder of a confocal microscope adapted for live-cell imaging.
72. Apply one drop of immersion oil type N on a 63× (NA 1.4) oil objective. The microscope should be prewarmed and atmospherically stabilized at 37°C and 5% CO_2_.
73. Select the appropriate laser lines and settings (intensity, interval etc), depending on the purpose of the experiment, the cell type and stimulus, expression levels of the fluorescent protein investigated and the intensity of the used fluorophore, and the type of adhesion structure to be analyzed.

***Note:*** In the experiment shown in Figure 7, we have used a 488 nm laser for illumination and an emission range of 500–550 nm for detection of β4-eGFP. Fluorescent and differential interference contrast (DIC) images were recorded every 5 min for 12 h.

74. For multi-position imaging, select xy positions using a linearly encoded motorized stage (Marzhauser) and save to positions list. Attempt to image several different fields per condition.

***Note:*** Use of the hardware autofocus control AFC may be necessary (troubleshooting 6). This option is called Definite Focus on Zeiss microscopes and Perfect Focus on Nikon machines.

75. Start the experiment to image a couple of frames in the absence of EGF.
76. 30 min after starting the experiment, very carefully add EGF (50 ng/mL).
77. Resume imaging for the desired time.

***Note:*** When comparing different conditions, capture images with the same settings for all conditions (first optimize settings on the control condition).

***Note:*** BSL-2 imaging facilities are required in case imaging is performed shortly after transduction.

***Note:*** The normal behavior of adhesion complexes, as well as proper capture of their dynamics, is influenced by many different factors (troubleshooting 7).

***Note:*** Risks of live-cell fluorescent imaging include signal bleaching and phototoxicity, which can be minimized with a number of approaches (troubleshooting 8).

Methods video S4. Dynamics of HDs, related to steps 52-67 of “live imaging of HDs using TIRF microscopy” Time-lapse movie of PA-JEB keratinocytes stably transduced with GFP-β4 and imaged using a Leica SP8 confocal set-up. Magnification 63× oil objective, interval 5 min, total time 690 min. See also Figure 7.

### Fluorescence recovery after photobleaching (FRAP)

**Timing: 1–2 days**

**Figure 8:**
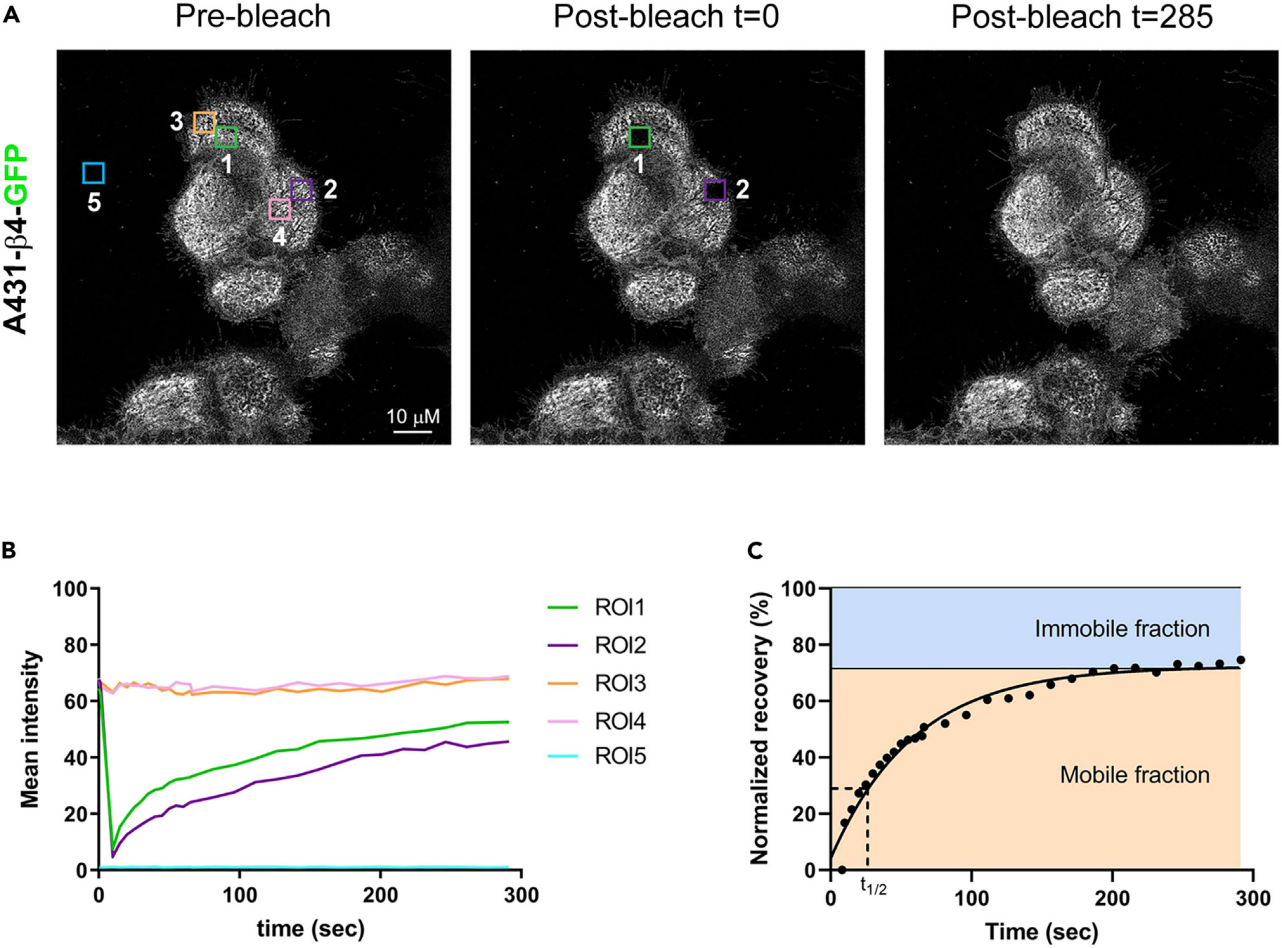
FRAP analysis of the dynamics of the HD integrin α6β4 in A431 cells (A) Confocal images before and after photobleaching of β4-GFP stably expressed in A431 cells and localized to HD-like structures. Two square regions of β4-GFP were selected (RO1 and ROI2) and photo-bleached through a high-power laser excitation. The recovery of fluorescence in the bleached area was monitored by taking images every 15 s for 5 min as described in fluorescence recovery after photobleaching (FRAP). To correct for photobleaching during this period, time-lapses of separate regions (ROI3 and ROI4) with identical acquisition settings to those used during the FRAP experiments were acquired. Time-lapses of a region (ROI5) outside the cells did not reveal any fluorescence. (B) Time-course of changes in fluorescence intensity in the bleached regions (ROI1 and ROI2), the surroundings (ROI4 and ROI5), and background (ROI3). (C) After background subtraction, compensation for imaging-induced photobleaching, and normalization, the recovery was fit with a single exponential curve (one-phase association) to calculate the half-time of recovery. Note that in this example, the fit was performed on the averaged normalized FRAP curves of only two ROI’s, but a combination of multiple ROIs from independent experiments is recommended.

This method describes how to analyze the dynamic behavior of proteins in adhesion complexes with FRAP, which relies on photobleaching of a fluorescent protein in selected ROI’s and time-lapse recording of fluorescence recovery as a parameter for protein mobility into the bleached area. As an example we here look at the mobility of the HD integrin α6β4 in A431 cells, which were stably transduced with β4-eGFP as described above (Figure 8). Bleaching and recording of fluorescence recovery were performed on a Leica SP5 laser scanning confocal system.^33^

78. Seed A431 cells on large circular coverslips (24 mm diameter) in DMEM/FCS.
79. Refresh the medium approximately 1 h before starting to set up the microscope.
80. Secure coverslip in an Attofluor™ Cell Chamber (Thermo Fisher Scientific).
81. Apply one drop of immersion oil type N on the 63× Plan Apochromat (NA 1.4) oil objective. The microscope should be prewarmed and atmospherically stabilized at 37°C and 5% CO_2_.
82. Select the appropriate laser lines (in this case for excitation at 488 nm) and settings (intensity, time interval, etc), depending on the expression levels and dynamics of the protein investigated, as well as cell type and stimuli (for instance specific growth factors).
83. Record 2 images of selected ROIs as pre-bleaching controls and 1 image as background control. Here we use the FRAP wizard in Leica Microsystems LAS X.
84. Switch to high-laser power for bleaching the selected ROI. Here we used a laser power of 100%, which corresponds to 3.5 mW, during 3.8 s.

***Note:*** Settings in this step and others in FRAP will need to be tested for your sample - for instance the ROI may be scanned several times for the bleaching step.

85. Record a post-bleaching time-lapse series (here: every 15 s during 5 min) to monitor fluorescence recovery.

***Note:*** In the experiment shown in Figure 8, we have used a detection range of 500–550 nm. Pinhole size was Airy 1, scan speed 400 Hz, and 2 frame averaging was employed.

86. For analysis, subtract the background fluorescence and if necessary, correct for the loss of fluorescence due to bleaching during image capture from the measured intensities. Generate curves. Normalize the curves to the mean value before bleaching.

***Note:*** In the experiment shown in Figure 8, no loss of fluorescence was observed for ROI’s 3 and 4 and therefore correction of the recovery values was not necessary.

***Note:*** Data can be used to calculate mobile fraction and recovery time (Figures 8B and 8C).

***Note:*** The dynamics of the protein under investigation may vary depending on its subcellular localization. Therefore, select multiple ROI’s per cell and perform analysis of multiple cells per condition (Figure 8).

### Assessment of integrin endocytic trafficking by microscopy

**Timing: 2-3 days**

In addition to imaging the behavior of fluorescently tagged integrins, integrin behavior can be followed using live-cell labeling with antibodies. This will allow live-cell imaging of integrin movement through the membrane, for instance of ligand-bound integrins along FN fibrils,^34^ or assessment of integrin endocytosis and recycling by pulse-chase assays.^21^^,^^35^^,^^36^ Here we describe a protocol for the synchronized endocytosis of β1 and β4 integrins in human keratinocytes and visualization by confocal laser scanning microscopy.

87. Culture cells in appropriate medium as described in cell culture and passage and on wells coated with appropriate ECM protein if necessary, as described in step 3 of prepare buffers, cell culture media, and substrates.
88. Seed cells on coated circular coverslips (12 mm diameter) in a 24 well plate in culture medium and place at 37°C and 5% CO_2_.
89. The next day, wash the cells with ice-cold serum-free medium in the cold room.
90. Label cell-surface integrins by incubating the cells with primary antibodies directed against integrin ectodomains and directly conjugated with fluorophores, diluted in ice-cold serum-free medium. Here we used antibody TS2/16 (conjugated to CY5) to label β1 and antibody 439-9B (conjugated to FITC) to label β4 (both at 1 μg/mL). Labeling should occur for 1 h on ice and in the cold room.

***Note:*** It is imperative to keep the cells cold to inhibit direct internalization of labeled integrins, which occurs at higher temperatures.

***Note:*** Use small coverslips and well sizes as this will reduce the volume of medium -and thus the amount of antibody- required. Antibodies against integrins can be used in concentration ranges 0.5–10 μg/mL roughly. Determine the optimal concentration first experimentally.

***Note:*** The choice of antibody may greatly affect results. Some antibodies react exclusively or preferentially with either active or inactive integrins, and these have very different kinetics of internalization, recycling, and degradation.^1^^,^^35^ In addition, antibodies can have functional effects on integrins (some of which related to the stabilization or alteration of specific integrin conformations) and this can affect integrin localization and behavior.^34^^,^^37^^,^^38^

**Figure 9:**
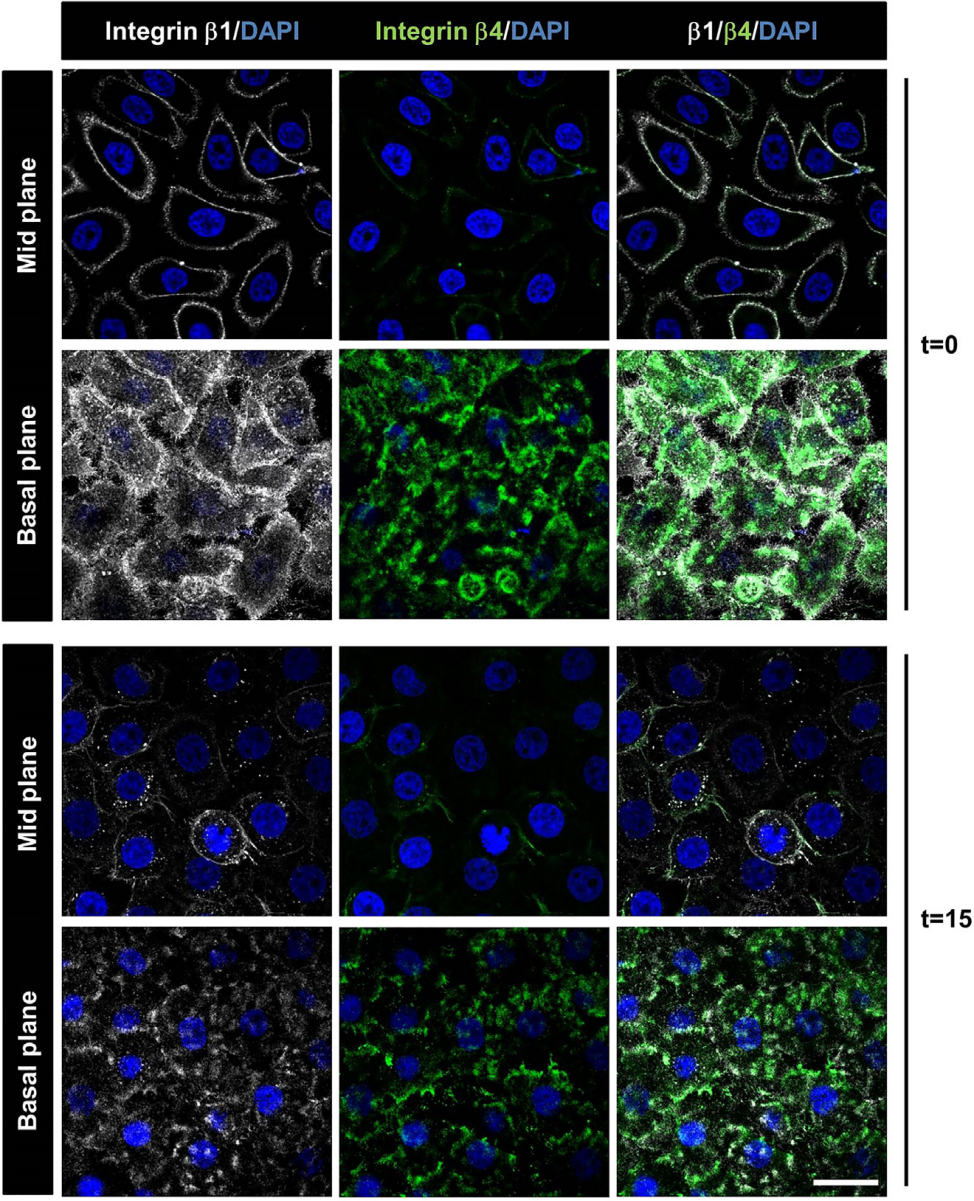
Pulse-chase assay for assessment of integrin internalization by confocal microscopy Confocal images showing the distribution of β1 integrins (pseudocolored *gray*) and integrin α6β4 (*green*) at the basal cell-surface (basal plane) and the lateral cell-surfaces (mid plane) in human PA-JEB/β4 keratinocytes. Labeling of β1 integrins and α6β4 occurred using fluorescent antibodies directed against integrin ectodomains at 4°C. Thereafter the cells were either directly fixed (t = 0) or after allowing integrin internalization for 15 min at 37°C (t = 15). Z-stacks were acquired as described in assessment of integrin endocytic trafficking by microscopy. Note that integrin β4 is much less abundant at the lateral cell-surfaces than β1 (mid plane, t = 0), and that there is also much less β4 internalization compared to β1 after 15 min (mid plane, t = 15). Bar, 40 μm.
92. Place the other coverslips at 37°C in serum-free medium to allow internalization of labeled cell-surface integrins.

***Note:*** As growth factors induce rapid recycling of internalized integrins, endocytosis is performed in the absence of serum to stimulate accumulation and retention of integrins in intracellular compartments.^39^

91. Transfer 1 or 2 coverslips directly after labeling to a well containing 4% PFA. These coverslips will reveal integrin distribution at the cell-surface before the onset of internalization (t = 0) (Figure 9).

93. Take out coverslips at appropriate time-points after inducing endocytosis (for instance 30 min) and transfer them to 4% PFA.

***Note:*** It is important to first determine the optimal time-points to observe robust integrin internalization for each cell type/integrin/condition as different integrins have very different kinetics of internalization and recycling,^40^^,^^41^^,^^42^ and in addition this may depend on cell type. In the example here, we observe robust β1 integrin internalization in 15 min in keratinocytes, while α6β4 hardly internalizes in this time-frame (Figure 9).

94. After fixing the last coverslips, process further if desired (for instance to label nuclei, adhesion complex components, or intracellular compartments) as described in staining of cell-matrix adhesions for immunofluorescence imaging.
95. Mount coverslips on microscope slides as described in staining of cell-matrix adhesions for immunofluorescence imaging.
96. Image the coverslips by confocal microscopy as described in confocal imaging of cell-matrix adhesions in fixed cells.

***Note:*** Here we used a Leica DM8 SP8 set-up with fully motorized Marzhauser stage, and a Leica 63×/1,40 oil HC plan apochromatic CS objective (Figure 9). Excitation was at 405 nm for DAPI, 488 nm for integrin β4, and 633 nm for integrin β1, and z-stacks consisting of 12 images were acquired starting at the basal plane where adhesions were clearly visible, moving upward with step size 1 μm, performing bidirectional and sequential scanning at a speed of 200 Hz.

## Expected outcomes

The protocols described here are designed to fluorescently label and visualize integrin-dependent cell-matrix adhesion complexes in a variety of cell types and perform detailed quantitative analysis of their characteristics (size, morphology and number). In addition, we present protocols for FRAP, live-cell labeling, and time-lapse imaging, generating insight into the dynamic properties of adhesion complexes or individual adhesion complex components over time, including motility, assembly and disassembly (turnover), and lifetime.

## Limitations

Several technical problems can occur at different steps in the protocols described here, associated for instance with cell culture, quality of the labelings, live-cell imaging, or the segmentation and analysis of results. Potential solutions to these issues are detailed in the troubleshooting section below, as well as in the notes throughout the manuscript.

## Troubleshooting

### Problem 1

Cells grow slower than anticipated, have an unexpected phenotype or morphology, or adhere and/or spread poorly to the tissue culture substrates (step 3 of prepare buffers, cell culture media, and substrates, steps 4-10 of cell culture and passage).

### Potential solution

In general, cell proliferation, morphology and survival are best when cell cultures are not too sparse and not too dense. Furthermore, it is always better to use lower cell passages as cells may undergo changes in growth characteristics during long-term culture. To specifically enhance integrin-dependent cell adhesion and spreading, as well as cell morphology and proliferation, appropriate coating of tissue culture materials is crucial. The coating of choice will depend in part on the cell type used and the tissue culture medium; media with 10% FCS have abundant serum components like FN and vitronectin, which supports cell adhesion and spreading of many cell types. More specialized cells grown in culture media with lower amounts of FCS may require pre-coating with matrix proteins, such as collagens or laminins for keratinocytes. Gelatin is routinely used to enhance adhesion of HUVECs.

### Problem 2

The number of integrin-dependent adhesion complexes is lower than anticipated and/or they have an unexpected appearance (step 3 of prepare buffers, cell culture media, and substrates, steps 4-10 of cell culture and passage).

### Potential solution

As for the previous point, it is crucial to maintain cells on the right matrix. For instance, vitronectin promotes the assembly of FAs and flat clathrin lattices, while FN promotes the formation of FAs and FBs. Laminin-332 supports the assembly of HDs and HD-like adhesions, as well as tetraspanin-enriched microdomains, containing integrin α3β1 in complex with CD151. Furthermore, because many cell types can rapidly cover the substrate with their own matrix (Figure 2), the type of adhesion complex that is formed and the integrins that are engaged are greatly influenced by the endogenously synthesized and deposited matrix. Furthermore, soluble factors in the culture medium can greatly affect the formation or dissolution of cell-matrix adhesions or promote cell migration, and actively migrating cells have different adhesion complexes than stationary cells. The type and appearance of cell-matrix complexes will further depend on which (combination of) integrins are expressed at the cell-surface, and it is recommended to identify the predominant integrins on the surface of a given cell type by methods such as flow cytometry. In many cell types, adhesions such as FAs tend to decrease in number with increasing cell density. Finally, the assembly of some complexes such as HDs and HD-like adhesions in keratinocytes is promoted by high calcium concentrations.

### Problem 3

The titer of lentiviral particles for transduction is low (steps 40-47 of production of lentiviral particles for transduction).

### Potential solution

Low titers are often the result of poor transfection efficiency of HEK293T cells due to compromised cell adhesion/spreading and/or proliferation (HEK393T cells are best transfected at a density of 50%–80%). This could be improved by coating the tissue culture dishes as described in step 3 of prepare buffers, cell culture media, and substrates. Moreover, low-passage HEK293T cells produce a higher titer than high-passage cells, and viral supernatants can be concentrated using PEG 6000 (Kutner et al., 2009). Finally, best results are obtained using freshly harvested supernatant, although supernatants can be stored at −80°C for weeks, months, or even longer.

### Problem 4

Adhesions are not clearly visible and/or there is high background staining (steps 5–18 of staining of cell-matrix adhesions for immunofluorescence imaging and steps 29–31 of confocal imaging of cell-matrix adhesions in fixed cells).

### Potential solution

The quality of cell-matrix adhesion labeling depends on a number of factors, including the antibody used, the relative abundance of the target protein and of adhesion complexes in general, the cell type, and the used culture conditions. It is recommended to try several different antibodies against typical adhesion proteins. Of note, some antibodies require a fixative other than PFA, such as MeOH. Poor labeling resulting from low numbers of adhesion complexes may be improved by changing the used matrix and/or culture conditions, as described in the previous point. Nonspecific staining may also be decreased by increasing the time of blocking or the use of alternative blocking solutions, containing for example bovine serum albumin, serum, or fish skin gelatin. Moreover, signal amplification methods such as tyramide signal amplification enable the visualization of low-abundance proteins. The signal-to-noise ratio of confocal images is further improved by using a low scanning speed for longer pixel dwell time (or a greater number of averaging), and capturing images at sufficient resolution (1024 × 1024 or higher). Moreover, line or frame averaging during acquisition will increase the signal-to-noise ratio. It is possible that adhesions at the basal cell surface are obscured by a strong cytoplasmic signal in the focal planes above, which can be solved by capturing only the basal plane using TIRF microscopy. Finally, images can be improved post-acquisition by enhancing contrast and subtracting background in software such as Fiji.

### Problem 5

Individual adhesions are hard to distinguish, complicating quantification (step 42 of automated analysis of cell-matrix adhesions in fixed cells).

### Potential solution

Segmentation of individual adhesions for analysis is a common problem and generally works best on clearly defined (discrete) structures. Therefore we use our macro here as an example to analyze FAs, which are usually more discrete structures, as compared to HDs and HD-like adhesions, for which it is impossible to define boundaries between individual adhesions. The macro performs thresholding and watershed to achieve FA segmentation. Still, it is possible that many small FAs appear as a large cluster or a continuous belt, particularly at the cell periphery in some cell types (Figure 4). Alternatively, watershed may arbitrarily increase the number of FAs. Improvement of FA staining or imaging at higher resolution could result in better identification of individual FAs. In case segmentation of individual adhesion remains problematic, results could be expressed by the total cell area occupied by adhesions (adhesive area).

### Problem 6

Focus drift occurs in time-lapse movies (live imaging of FAs using TIRF microscopy, live imaging of FBs using widefield microscopy, and live imaging of HDs using TIRF microscopy).

### Potential solution

Focus drift during time-lapse imaging is a significant problem that can have several causes. When performing multi-position imaging, it is advised to first create a positions list and then check each position at least once to make sure all positions are still in focus. Many microscope systems are provided with an autofocus function, which may help to prevent focus loss in time-lapse series. Moreover, water or dry objectives are better for multi-position imaging than oil immersion objectives, as the oil will be distributed all over the plate/coverslip while the microscope stage changes positions, increasing the risk of focus loss. It may also be helpful to make a small stack of images (3–4) at each position to increase the chance of having one of them in focus. TIRF imaging is extremely sensitive to minor fluctuations in air current or temperature, and it is essential that the microscope is placed on a solid anti-vibration table, and to temperature-equilibrate the plate/chamber or coverslip containing the cells and imaging medium prior to onset of the experiment. Several solutions exist that may correct drift in movies. We have here used the Fiji plugin “*Linear Stack Alignment with SIFT*”, based on the SIFT algorithm (https://imagej.net/plugins/linear-stack-alignment-with-sift).^24^

### Problem 7

Adhesion behavior is less dynamic than expected in time-lapse movies (steps 49-51 of live imaging of FAs using TIRF microscopy, steps 63-67 of live imaging of FBs using widefield microscopy, and steps 74-75 of live imaging of HDs using TIRF microscopy).

### Potential solution

As with any live-cell experiment, it is important that conditions are as close to normal physiological circumstances as possible, meaning that proper temperature, humidity, CO_2_/O_2_ and pH of the medium are maintained. The dynamics of adhesion complexes depend strongly on the type of adhesion complex, the cell type, and the culture conditions. Cell migration is a key driver of adhesion assembly and disassembly, and FA turnover is therefore strongly promoted by pro-migratory stimuli, including growth factors. A more robust effect may be achieved by first depriving the cells of growth factors for a couple of hours prior to the start of the experiment, and then re-stimulate them with normal culture medium containing growth factors or a defined factor of interest. Adhesion dynamics are also strongly regulated by agents that induce cell contraction/retraction such as thrombin, used in the example we provide here (Figure 6), or relaxation (such as blebbistatin). Moreover, it is important to first determine the proper time interval between images; dynamics will be missed when using too long intervals.

### Problem 8

The signal is bleaching fast, cells look unhealthy, cells do not behave as expected or die (steps 49-51 of live imaging of FAs using TIRF microscopy, steps 63-67 of live imaging of FBs using widefield microscopy, and steps 74-75 of live imaging of HDs using TIRF microscopy).

### Potential solution

Illumination during live imaging can cause signal bleaching due to degradation of fluorophores, and may compromise cell health and viability due to phototoxicity. Solutions may include imaging at lower laser power and increasing the interval between consecutive images (thus reducing the number of times the sample is exposed), or use of resonant scanning (on a confocal microscope). In addition, the use of other systems like light-sheet microscopes could also resolve these problems.

## Resource availability

### Lead contact

Further information and requests for resources and reagents should be directed to and will be fulfilled by the lead contact, Coert Margadant (c.margadant@biology.leidenuniv.nl), or Arnoud Sonnenberg (a.sonnenberg@nki.nl).

### Materials availability

This study did not generate new unique reagents.

## Data Availability

The ImageJ macro “*FA analyzer*” is available on GitHub: https://github.com/agclark12/FA_analyzer or Zenodo: https://doi.org/10.5281/zenodo.808911.
